# Spectrum of activity and mechanisms of azole–bisphosphonate synergy in pathogenic *Candida*

**DOI:** 10.1128/spectrum.00121-24

**Published:** 2024-05-02

**Authors:** Aidan Kane, Hue Dinh, Leona Campbell, Amy K. Cain, David Hibbs, Dee Carter

**Affiliations:** 1 School of Life and Environmental Sciences and the Sydney Institute for Infectious Diseases, The University of Sydney, Sydney, New South Wales, Australia; 2 School of Natural Sciences, ARC Centre of Excellence in Synthetic Biology, Macquarie University, Sydney, New South Wales, Australia; 3 School of Pharmacy, The University of Sydney, Sydney, New South Wales, Australia; Institut Pasteur, Paris, France

**Keywords:** *Candida albicans*, azole, drug synergy, bisphosphonate, zoledronate, fluconazole, *Candida glabrata*, mevalonate pathway

## Abstract

Candidiasis places a significant burden on human health and can range from common superficial vulvovaginal and oral infections to invasive diseases with high mortality. The most common *Candida* species implicated in human disease is *Candida albicans*, but other species like *Candida glabrata* are emerging. The use of azole antifungals for treatment is limited by increasing rates of resistance. This study explores repositioning bisphosphonates, which are traditionally used for osteoporosis, as antifungal synergists that can improve and revitalize the use of azoles. Risedronate, alendronate, and zoledronate (ZOL) were tested against isolates from six different species of *Candida*, and ZOL produced moderate antifungal activity and strong synergy with azoles like fluconazole (FLC), particularly in *C. glabrata*. FLC:ZOL combinations had increased fungicidal and antibiofilm activity compared to either drug alone, and the combination prevented the development of antifungal resistance. Mechanistic investigations demonstrated that the synergy was mediated by the depletion of squalene, resulting in the inhibition of ergosterol biosynthesis and a compromised membrane structure. In *C. glabrata*, synergy compromised the function of membrane-bound multidrug transporters and caused an accumulation of reactive oxygen species, which may account for its acute sensitivity to FLC:ZOL. The efficacy of FLC:ZOL *in vivo* was confirmed in a *Galleria mellonella* infection model, where combinations improved the survival of larvae infected with *C. albicans* and *C. glabrata* to a greater extent than monotherapy with FLC or ZOL, and at reduced dosages. These findings demonstrate that bisphosphonates and azoles are a promising new combination therapy for the treatment of topical candidiasis.

**IMPORTANCE:**

*Candida* is a common and often very serious opportunistic fungal pathogen. Invasive candidiasis is a prevalent cause of nosocomial infections with a high mortality rate, and mucocutaneous infections significantly impact the quality of life of millions of patients a year. These infections pose substantial clinical challenges, particularly as the currently available antifungal treatment options are limited in efficacy and often toxic. Azoles are a mainstay of antifungal therapy and work by targeting the biosynthesis of ergosterol. However, there are rising rates of acquired azole resistance in various *Candida* species, and some species are considered intrinsically resistant to most azoles. Our research demonstrates the promising therapeutic potential of synergistically enhancing azoles with non-toxic, FDA-approved bisphosphonates. Repurposing bisphosphonates as antifungal synergists can bypass much of the drug development pipeline and accelerate the translation of azole–bisphosphonate combination therapy.

## INTRODUCTION

Candidiasis is an infection caused by the overgrowth of *Candida*, a genus containing some of the world’s most important fungal pathogens. Non-invasive candidiasis involves infection of the vulvovaginal and oral mucosa and is responsible for more than 3.6 million annual hospital visits in the United States and significant morbidity for immunocompromised patients (1, 2). Invasive candidiasis occurs when *Candida* cells breach the mucosa and disseminate via the bloodstream to peripheral tissues (3). Invasive candidiasis places a significant burden on public health as it is associated with long periods of intensive care unit occupancy and has a mortality rate of up to 24% (4). Worldwide, it is estimated that invasive candidiasis causes more than 50,000 deaths each year (5).

*Candida albicans* is the most common species causing candidiasis, and it colonizes the mucosa of 35%–80% of healthy adults (6). There are 15 known species of non-*albicans Candida* that cause disease in humans, and the most common are *Candida glabrata, Candida parapsilosis,* and *Candida tropicalis* (7). Recurring infections caused by *C. glabrata* are increasing significantly due to the widespread use of immunosuppressive therapies and broad-spectrum antimicrobials (8). *Candida albicans* and *C. glabrata* can both be transmitted nosocomially due to their ability to form biofilms on host tissues and medical devices (9). These biofilms are difficult to treat as they are resistant to most traditional antifungals, and many agents for managing bacterial biofilms are ineffective (10).

Invasive candidiasis is predominantly treated with intravenous echinocandins, but other antifungals like amphotericin B (AMB) and fluconazole are also used (11). Oral candidiasis is systemically treated with fluconazole, but due to increasing rates of resistance, itraconazole and ketoconazole are often employed instead (12). Azole antifungals are an important part of the clinical toolbox and work by inhibiting Erg11, preventing the biosynthesis of ergosterol. More than 5% of modern *C. albicans* isolates are azole-resistant, and outbreaks of fluconazole-resistant *C. parapsilosis* have been occurring worldwide since 2018 (13, 14). Emerging pathogen *Candida krusei* and some isolates of *C. tropicalis* are intrinsically azole-resistant due to binding site mutations in Erg11 (15–17). *Candida glabrata* has the highest rates of resistance among all species of *Candida,* primarily due to its propensity to express membrane-bound active efflux pumps (18).

Due to the increasing emergence of drug-resistant cases of candidiasis, there is an urgent need for new antifungals. One promising approach to antimicrobial development is the design and discovery of compounds that synergistically improve azole antifungals (19). Combining drugs improves efficacy, decreases the required dose, and overcomes and prevents the development of antimicrobial resistance, and if commercially available FDA-approved drugs can be used, this substantially expedites the development pipeline (20, 21). Bisphosphonates are a class of drugs used to treat osteoporosis and other low-bone density disorders, and they have also been shown to have antiparasitic, anticancer, and immunostimulatory effects (22–26). Bisphosphonates inhibit farnesyl pyrophosphate synthetase (FPPS), which affects geranylgeranyl transferase, protein prenylation, and squalene synthesis (22).

In our previous work using a comprehensive suite of *Cryptococcus* isolates, we showed that three FDA-approved bisphosphonates targeted the mevalonate pathway, inhibiting squalene synthesis and resulting in antifungal activity. As squalene is a critical precursor to ergosterol biosynthesis, which is the target of azoles, we found that bisphosphonates synergize with fluconazole by operating on a closely related biochemical pathway (27). In the current study, we extend this analysis to a collection of *Candida* isolates. Our results suggest that combination therapy with azoles and bisphosphonates is a promising therapeutic lead with potential clinical applications.

## RESULTS

### Amino-bisphosphonates have antifungal activity against *Candida* pathogens

Bisphosphonates were tested for antifungal activity against 46 isolates of the genus *Candida*. Minimum inhibitory concentrations (MICs) were obtained using CLSI broth microdilution methods (28). Non-amino-bisphosphonates etidronate and clodronate were not antifungal in any species at the highest concentration tested, 512 µg/mL (not shown). Amino-bisphosphonates risedronate (RIS), alendronate (ALN), and zoledronate (ZOL) showed antifungal activity with azole antifungals fluconazole (FLC), itraconazole (ITR), and ketoconazole (KET). MICs for each strain are detailed in Table S1 and summarized in Table 1. ZOL was the most active bisphosphonate across all species tested [MIC geometric mean (GM) = 34.33 µg/mL] and was significantly more active than RIS (GM = 63.44 µg/mL) (*P* < 0.0001) and ALN (GM = 55.07 µg/mL) (*P* < 0.0001). For simplicity, the amino-bisphosphonates will be referred to as bisphosphonates throughout this study.

**TABLE 1:** Mean MICs and minimum fungicidal concentrations (MFCs) of three azoles and three bisphosphonates for six clinically relevant *Candida* species

Species (no. of strains tested)		Geometric mean MIC/MFC ± SD (µg/mL)					
		FLC	ITR	KET	RIS	ALN	ZOL
*Candida albicans* (*n* = 11)	MIC	0.64 ± 0.51	0.18 ± 0.15	0.16 ± 0.29	329.39 ± 129.16	256 ± 152.43	145.19 ± 133.69
	MFC	2.92 ± 2.88	4.83 ± 9.53	2.27 ± 4.98	>256	>256	145.19 ± 133.69
*Candida glabrata* (*n* = 11)	MIC	30.05 ± 74.67	4.26 ± 9.61	3.76 ± 9.21	8 ± 17.62	5.15 ± 5.97	2.57 ± 2.58
	MFC	154.64 ± 165.56	19.33 ± 11.93	19.33 ± 11.73	82.35 ± 150.45	34.08 ± 50.1	10.96 ± 10.18
*Candida parapsilosis* (*n* = 6)	MIC	0.63 ± 0.66	0.09 ± 0.04	0.09 ± 0.19	362.04 ± 170.13	322.54 ± 170.13	64 ± 43.72
	MFC	3.56 ± 12.05	1.12 ± 0.84	2 ± 5.95	>256	322.54 ± 170.13	322.54 ± 170.13
*Candida krusei* (*n* = 5)	MIC	48.5 ± 17.53	1.74 ± 0.45	0.33 ± 0.14	>256	>256	294.07 ± 114.49
	MFC	>256	12.13 ± 5.37	8 ± 6.07	>256	>256	>256
*Candida tropicalis* (*n* = 7)	MIC	35.33 ± 48.66	5.94 ± 10.89	4 ± 6.81	420.01 ± 124.92	420.01 ± 124.92	128 ± 57.58
	MFC	256 ± 209.84	17.67 ± 11.99	17.67 ± 12.26	463.73 ± 96.76	>256	312.07 ± 160.46
*Candida dubliniensis* (*n* = 6)	MIC	0.4 ± 0.39	0.16 ± 0.38	0.04 ± 0.05	128 ± 87.44	128 ± 86.85	10.08 ± 10.63
	MFC	3.56 ± 7.37	1.41 ± 3.79	1.26 ± 3.03	406.37 ± 132.2	406.37 ± 156.77	57.02 ± 47.19

*Candida glabrata* and *Candida dubliniensis* were especially susceptible to RIS, ALN, and ZOL compared to the other species tested. *Candida glabrata* had exceptionally low MICs for RIS (GM = 8.00 µg/mL), ALN (GM = 5.15 µg/mL), and ZOL (GM = 2.57 µg/mL). *Candida dubliniensis* was less susceptible to RIS (GM = 128 µg/mL) and ALN (GM = 128 µg/mL) but was susceptible to ZOL (GM = 10.08 µg/mL). *Candida krusei* was the least bisphosphonate-susceptible species tested; RIS and ALN were unable to inhibit growth at 256 µg/mL, and ZOL had an MIC of 256 µg/mL in all but one strain. Bisphosphonate MICs had no significant correlation with azole resistance in *C. glabrata* (*r* = 0.1657, *P* = 0.6069), with ZOL being active against even highly azole-resistant isolates.

MFCs are detailed in Table S1 and summarized in Table 1. Bisphosphonates had limited fungicidal activity against most *Candida* species tested. ZOL was the most fungicidal bisphosphonate across all isolates (MFC GM = 59.7 µg/mL) and was significantly more fungicidal than RIS (GM = 111.9 µg/mL) (*P* < 0.0001) and ALN (GM = 64.22 µg/mL) (*P* < 0.0001). Across all species, ZOL MFCs were ~1.74-fold higher than ZOL MICs, and MFCs were 1.16- and 1.76-fold higher than MICs for ALN and RIS, respectively.

### Bisphosphonates synergize with azole antifungals and potentiate fungicidal activity in most species of *Candida*

**Fig 1:**
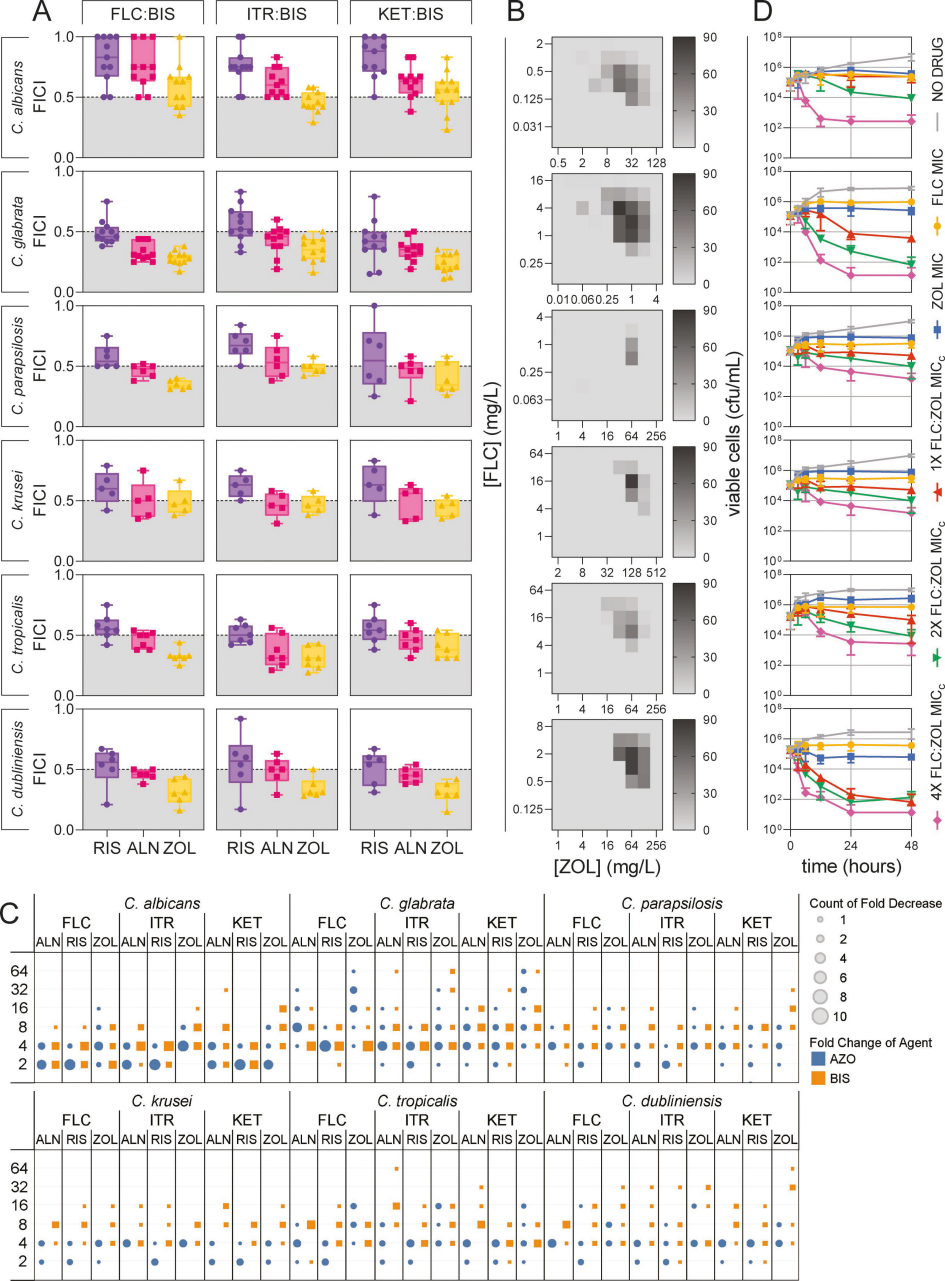
Synergy between three azole antifungals and three bisphosphonates in six different clinically relevant species of *Candida*. (**A**) FICIs for combinations of FLC, ITR, and KET with RIS, ALN, and ZOL. Each point represents the FICI for a single strain, calculated as the mean of three biological replicates. (**B**) Two-dimensional MacSynergy II surfaces produced from analysis of checkerboards for FLC:ZOL combinations indicate significant synergy in all species except *C. parapsilosis*. (**C**) Fold decrease for each drug when used separately (MIC) and in combination (MIC_c_). Azoles are shown as blue circles and bisphosphonates as orange squares. The size of each circle/square indicates the number of strains with each fold decrease, as detailed in Table S2. (**D**) Time-kill assays obtained by treating each *Candida* isolate with a no-drug control (1% DMSO) (gray line), FLC (yellow circles), or ZOL (blue squares) at the MIC described in Table S1, and FLC:ZOL combinations at one (orange triangles), two (green inverted triangles), and four (pink diamonds) times the MIC_c_ described in Table S2. Data presented are the means of four technical replicates of three biological replicates ± SD.

The synergy between each pairing of azoles (FLC, ITR, and KET) with bisphosphonates (RIS, ALN, and ZOL) was assessed with the checkerboard assay (29). The MICs of each drug when used in combination (defined here as MIC_c_) are detailed in Table S2. Fractional inhibitory concentration indices (FICIs) were calculated and are detailed in Table S2 and Fig. 1A. ZOL was the most synergistic bisphosphonate when combined with all three azoles. Across all species, FLC:ZOL was the most synergistic azole–bisphosphonate combination. FLC:ZOL was synergistic in 85% of strains, ITR:ZOL in 79%, and KET:ZOL in 74%. FLC:ALN was synergistic in 70% of strains, ITR:ALN in 59%, KET:ALN in 63%, FLC:RIS in 39%, ITR:RIS in 33%, and KET:RIS in 39%.

Combinations of azoles and bisphosphonates were most synergistic in *Candida glabrata.* Across the strains tested within each species, FLC:ZOL was synergistic in 45.5% of *C. albicans* strains, 80% of *C. krusei* strains, and 100% of *C. glabrata, C. parapsilosis, C. tropicalis,* and *C. dubliniensis* strains. The mean ± SD FICIs for all azole–bisphosphonate combinations were 0.67 ± 0.062 in *Candida albicans*, 0.38 ± 0.058 in *C. glabrata,* 0.5 ± 0.0545 in *C. parapsilosis*, 0.52 ± 0.037 in *C. krusei*, 0.439 ± 0.066 in *C. tropicalis,* and 0.439 ± 0.085 in *C. dubliniensis*. The FICIs across all azole–bisphosphonate combinations were significantly lower in *C. glabrata* than *C. albicans* (*P* < 0.0001), *C. parapsilosis* (*P* < 0.0001), *C. krusei* (*P* < 0.0001), and *C. tropicalis* (*P* = 0.0481), but not *C. dubliniensis* (*P* = 0.1284).

Drug interaction checkerboards were also analyzed with MacSynergy II to obtain Bliss Independence synergy volumes and dose–response heatmaps (Fig. 1B) (30). Using this model of synergy, FLC:ZOL was strongly synergistic in *C. albicans* SC5314 (366.57 µM^2^%), *C. glabrata* CBS138 (760.01 µM^2^%), and *C. dubliniensis* M230642 (526.09 µM^2^%). Moderate synergy was observed in *C. tropicalis* M230640 (211.40 µM^2^%) and *C. krusei* ATCC6258 (201.75 µM^2^%). FLC:ZOL was minimally synergistic in *C. parapsilosis* ATCC22018 (88.79 µM^2^%).

The fold decrease for drugs used in combination (MIC_c_) compared to drugs used alone (MIC) is detailed in Table S2 and summarized in Fig. 1C. These fold decreases show that although some combinations may not meet the threshold to be considered synergistic, bisphosphonates can significantly lower the concentration of FLC, ITR, and KET required to inhibit growth. The GM fold decrease for FLC in FLC–bisphosphonate combinations was 4.26 for FLC:RIS, 9.67 for FLC:ALN, and 21.93 for FLC:ZOL. The GM fold decrease for ITR in ITR–bisphosphonate combinations was 3.31 for ITR:RIS, 4.83 for ITR:ALN, and 6.21 for ITR:ZOL. For KET, the GM fold decrease when used in KET–bisphosphonate combinations was 6.22 for KET:RIS, 5.48 for KET:ALN, and 16 for KET:ZOL. As FLC:ZOL combinations displayed very significant, broad-spectrum synergy and dramatically decreased inhibition even in azole-resistant strains, FLC:ZOL combinations were investigated further in this study.

Time-kill assays showed that while FLC and ZOL were fungistatic at MIC, combinations of FLC:ZOL potentiated fungicidal killing in *Candida* (Fig. 1D). FLC:ZOL at the MIC_c_ was slightly fungicidal in *Candida glabrata,* with a 30.5-fold decrease in viable cell count over 48 hours. FLC:ZOL at the MIC_c_ was more fungicidal in *C. dubliniensis*, with a 2,920-fold decrease. At 4× the MIC_c_, all species tested experienced significant fungicidal killing after 48 hours. The fractional fungicidal concentration indices (FFCIs) confirmed the fungicidal nature of FLC:ZOL combinations (Table S3).

### Azoles and bisphosphonates have limited antibiofilm activity alone*,* but azole–bisphosphonate combinations synergistically inhibit biofilms of *Candida*

Bisphosphonates alone were able to inhibit biofilms of *C. glabrata* and *C. dubliniensis* with sessile MIC_80_ values of 128–1,024 µg/mL, but these were not able to inhibit the other *Candida* species (Table 2).

**TABLE 2:** Sessile MIC_80_ (SMIC_80_) and FICI (SFIC) values for combinations of azoles and bisphosphonates in biofilms formed by various *Candida* species

Species	Strain	FLC						SFICI^*a*^								
			SMIC_80_ (μg/mL)					FLC:BIS			ITR:BIS			KET:BIS		
			ITR	KET	RIS	ALN	ZOL	RIS	ALN	ZOL	RIS	ALN	ZOL	RIS	ALN	ZOL
*C. albicans*	SC5314	512	256	64	>1,024	>1,024	>1,024	1.13	0.75	*0.50*	1.50	0.83	0.60	2.00	1.13	0.63
*C. glabrata*	CBS138	>1,024	256	256	512	256	128	*0.42*	*0.38*	*0.25*	0.82	0.63	*0.50*	*0.45*	*0.31*	*0.25*
*C. parapsilosis*	ATCC22018	256	64	16	>1,024	>1,024	>1,024	0.83	*0.50*	*0.42*	1.00	0.82	0.75	1.50	1.06	0.63
*C. krusei*	ATCC6258	>1,024	128	256	>1,024	>1,024	>1,024	0.79	0.63	0.56	0.83	0.63	*0.50*	1.25	1.06	0.75
*C. tropicalis*	M230640	>1,024	>1,024	>1,024	>1,024	>1,024	>1,024	0.83	*0.50*	*0.38*	1.00	1.12	0.56	0.75	0.67	*0.50*
*C. dubliniensis*	M230642	256	64	32	1,024	256	256	0.67	0.60	*0.38*	0.79	0.83	*0.45*	0.71	*0.38*	*0.31*

^*a*^ Synergistic combinations (SFICI ≤ 0.5) are shown in italics.

SFICIs were calculated by comparing the SMIC_80_ of drugs alone with the SMIC_c_ of drugs in combination (Table 2). With the exception of *C. krusei*, FLC:ZOL combinations were the most synergistic. The average FLC:ZOL SFICI across all species was 0.42, significantly lower than FLC:ALN (mean SFICI = 0.56; *P* = 0.046) and FLC:RIS (mean SFICI = 0.78; *P* = 0.0032). For *C. krusei* biofilms, the most synergistic combination was ITR:ZOL (SFICI = 0.50). In biofilms formed by *C. glabrata,* 77.8% of azole–bisphosphonate combinations were synergistic, compared to 11.1% for *C. albicans* and *C. krusei,* 22.2% for *C. parapsilosis*, 33.3% for *C. tropicalis,* and 44.4% for *C. dubliniensis*.

### Combinations of fluconazole and zoledronate prevent the development of antifungal resistance in all *Candida* species

**Fig 2:**
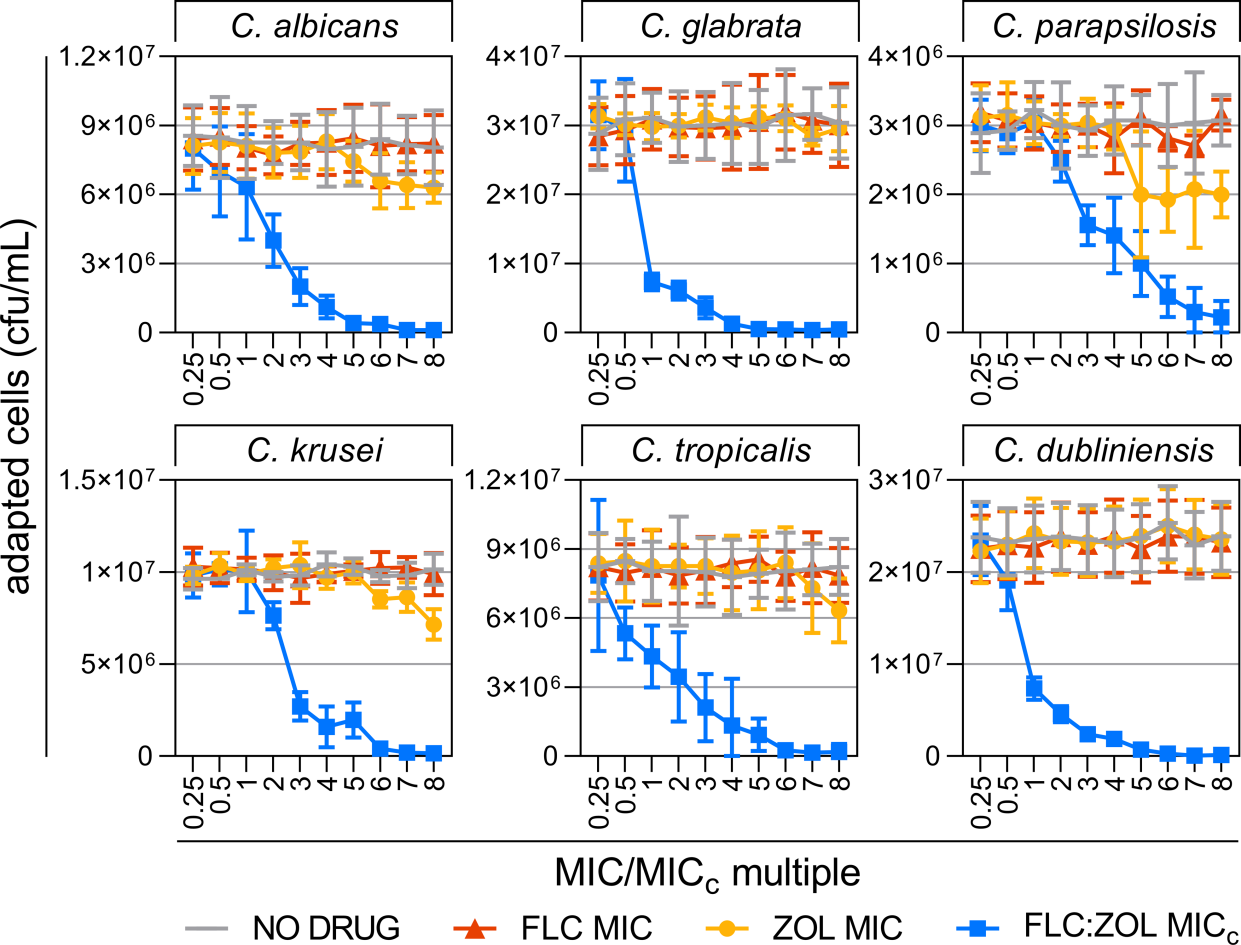
Combinations of azoles and bisphosphonates prevent the development of antifungal resistance. Actively growing cultures of *C. albicans* SC5314, *C. glabrata* CBS138, *C. parapsilosis* ATCC22018, *C. krusei* ATCC6258, *C. tropicalis* M230640, and *C. dubliniensis* M230642 were passaged through increasing concentrations of FLC (orange triangles), ZOL (yellow circles), FLC:ZOL combinations (blue squares), and a no-drug control (1% DMSO) (gray line), beginning at subinhibitory doses and ending at eight times the MIC or MIC_c_. Data presented are the means of four technical replicates of three biological replicates ± SD.

One of the key advantages of combining antimicrobials is that it lowers the probability of susceptible cells developing resistance (31). When *Candida* cells were exposed to subinhibitory concentrations (0.25× MIC) of FLC and ZOL, then propagated into solutions of increasing drug concentration, they became tolerant to extremely high concentrations of both drugs (Fig. 2). When exposed to subinhibitory concentrations (0.25× MIC_c_) of FLC:ZOL and propagated in the same way, very few cells grew beyond 4–5× MIC_c_; the exception was *C. parapsilosis,* where some viability extended beyond 7× MIC_c_. Slightly reduced viability was observed at 5–8× ZOL MIC for *C. albicans, C. parapsilosis, C. krusei,* and *C. tropicalis* cells, possibly due to the osmotic effect of extremely high concentrations of ZOL.

### The antifungal effects of FLC:ZOL combinations are due to bisphosphonate-mediated inhibition of the mevalonate pathway

Due to its status as an emerging pathogen and prominent cause of recurring candidiasis and its substantial sensitivity to ZOL and FLC:ZOL synergy, *Candida glabrata* was investigated further to determine the mechanism/s underlying synergy. CBS138, an azole-susceptible reference strain, and M494893, a highly azole-resistant clinical isolate, were included. Reference strains *C. krusei* ATCC6258, which has reduced azole sensitivity and showed limited FLC:ZOL synergy, and *C. albicans* SC5314, which is azole-sensitive and with limited FLC:ZOL synergy, were included for comparison. The dosages of FLC, ZOL, FLC:ZOL, and any positive control compounds used in mechanistic experiments are detailed in Table 3.

**TABLE 3:** MIC and MIC_c_ values for FLC, ZOL, FLC:ZOL, and positive controls AMB and H_2_O_2_ as used in mechanistic experiments throughout this study

Species	Strain			FLC:ZOL MIC_c_			
		FLC MIC	ZOL MIC	FLC MIC_c_	ZOL MIC_c_	AMB MIC	H_2_O_2_ MIC^*a*^
*C. albicans*	SC5314	1	64	0.25	16	0.125	5.5
*C. glabrata*	CBS138	8	2	0.5	0.25	0.25	22
*C. glabrata*	M494893	256	4	8	0.5	0.5	11
*C. krusei*	ATCC 6258	32	256	8	128	0.25	1.38

^*a*^ H_2_O_2_ MICs are given in millimolar concentrations; all others are in microgram per milliliter.

**Fig 3:**
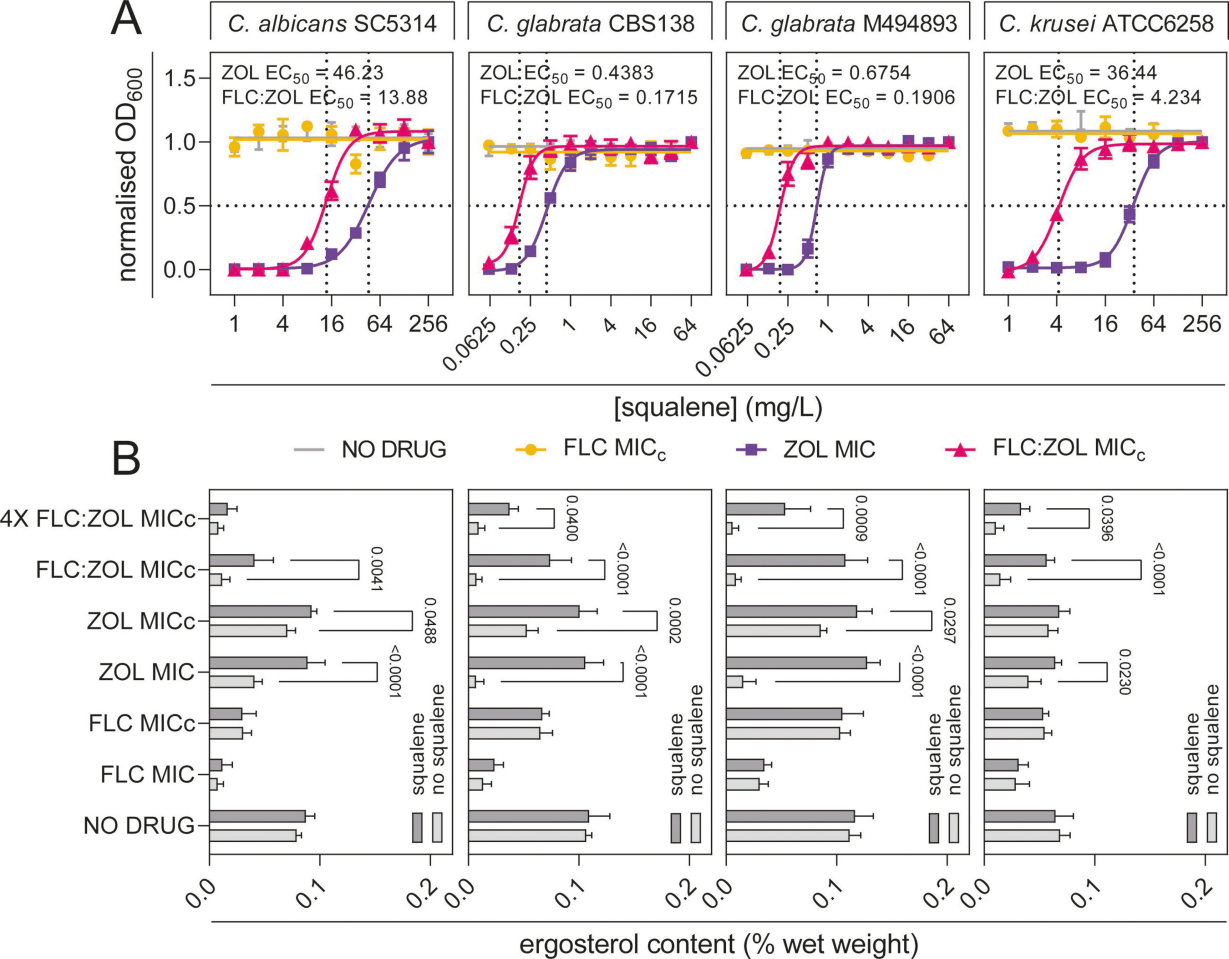
Bisphosphonates inhibit *Candida* by targeting the mevalonate pathway and depleting membrane ergosterol. (**A**) Squalene rescue assay; *C. albicans* SC5314, *C. glabrata* CBS138, *C. glabrata* M494893, and *C. krusei* ATCC6258 were treated with a no-drug control (1% DMSO) (gray lines), FLC at MIC_c_ (yellow circles), ZOL at MIC (purple squares), or FLC:ZOL at MIC_c_ (pink triangles). Exogenous squalene was added from 1 to 64 µg/mL for *C. glabrata* and 1 to 256 µg/mL for *C. albicans* and *C. krusei*. OD_600_ was normalized to a no-culture and a no-treatment control, and non-linear regression analysis was performed to obtain a dose–response curve. Dotted lines indicate the squalene rescue EC_50_s for ZOL and FLC:ZOL treatments. Data presented are the means of two technical replicates for each of three biological replicates ± SD. (**B**) Ergosterol content following drug treatment with and without the addition of squalene (128 µg/mL), calculated as a percentage of total wet weight of pelleted *Candida* culture. Data are the means of three technical replicates of each of three independent biological replicates ± SD.

To determine if impaired squalene synthesis was responsible for the synergy described above, increasing doses of squalene were added to cultures treated with ZOL and FLC:ZOL (Fig. 3A). Rescue of growth by exogenous squalene occurred in a dose-dependent manner for all strains. For ZOL, the concentration of squalene required for rescue was lower for the two strains of *C. glabrata* (EC_50_ range 0.1715–0.6754 µg/mL) than for either *C. albicans* or *C. krusei* (EC_50_ range 4.234–46.23 µg/mL), indicating that *C. glabrata* may be more sensitive to squalene deprivation. The rescue of treated cells suggests that the inhibition of the mevalonate pathway is a key antifungal mechanism for both ZOL alone and in combination with FLC in all species.

Inhibition of squalene synthesis by ZOL and FLC:ZOL treatment resulted in the depletion of ergosterol in the *Candida* membrane (Fig. 3B). In the absence of squalene supplementation, treatment with FLC at 1× MIC caused a significant decrease in ergosterol content in all four strains when compared to the no-drug control (*P* < 0.0001). ZOL at 1× MIC significantly reduced ergosterol content in *C. albicans* (*P* = 0.0002), *C. glabrata* CBS138 (*P* < 0.0001), *C. glabrata* M494893 (*P* < 0.0001), and *C. krusei* (*P* = 0.0039). FLC:ZOL treatment at 1× MIC_c_ resulted in a sharp decrease in ergosterol content in all four strains, compared to the no-drug control (*P* < 0.0001). There was no significant difference in ergosterol content between 1× MIC_c_ FLC:ZOL and FLC MIC treatments in *C. albicans*, *C. glabrata* M494893, or *C. krusei* (*P* range =0.4316–0.9982), but there was in *C. glabrata* CBS138 (*P* < 0.0001). The addition of exogenous squalene significantly increased ergosterol content in ZOL MIC-treated *C. albicans* (*P* < 0.0001), *C. glabrata* CBS138 (*P* < 0.0001), *C. glabrata* M494893 (*P* < 0.0001), and *C. krusei* (*P* = 0.0230), and this was also observed following treatment with FLC:ZOL at 1× MIC_c_ (*C. albicans*; *P* = 0.0041, *C. glabrata* CBS138, *C. glabrata* M494893, and *C. krusei*; *P* < 0.0001). The ergosterol content in squalene-supplemented cells treated with ZOL and FLC:ZOL at 1× MIC_c_ was not significantly different from the no-drug control in any strain, except for *C. glabrata* CBS138, where squalene supplementation following 1× FLC:ZOL treatment still resulted in reduced ergosterol content (*P* = 0.0041). Squalene supplementation was unable to restore ergosterol content in FLC-treated cells in any of the four strains tested or in *C. albicans* cells treated with FLC:ZOL at 4× MIC_c_. Treatment with FLC:ZOL at 4× MIC_c_ was so harmful in all four strains that squalene supplementation was unable to restore ergosterol content to the levels in the no-drug control (*P* range = 0.0048–<0.0001).

### Bisphosphonate-mediated ergosterol and squalene depletion results in reduced membrane rigidity and active efflux

**Fig 4:**
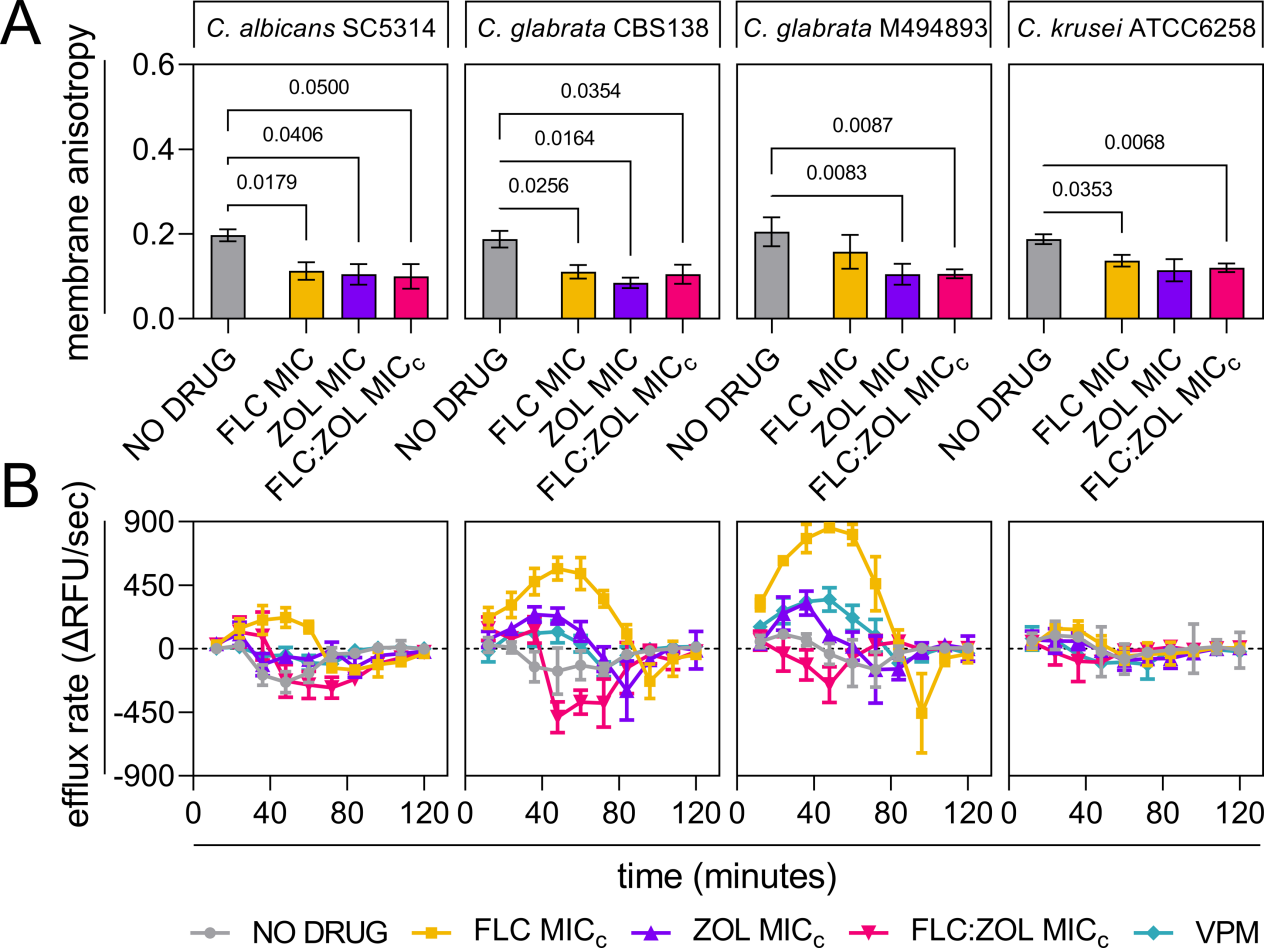
Bisphosphonate-mediated ergosterol depletion results in membrane hyperfluidity and the disruption of active efflux. (**A**) Membrane fluidity expressed as anisotropy, for *Candida* cells treated with a no-drug control (1% DMSO), FLC or ZOL at MIC, and FLC:ZOL at MIC_c_. Fluidity is inversely proportional to anisotropy. (**B**) Rates of active efflux of R6G from *Candida* cells treated with a no-drug control (1% DMSO) (gray circles), FLC (yellow squares), ZOL (purple triangles), and FLC:ZOL (pink inverted triangles), all at MIC_c_. A positive control of verapamil (VPM), a known pump inhibitor, was included at 2 mg/mL. Data presented are the means of three biological replicates ± SD.

The fluorescence anisotropy of membrane-bound 1-(4-trimethylammoniumphenyl)-6-phenyl-1,3,5-hexatriene *p*-toluenesulfonate (TMA-DPH) was measured to evaluate the effects of ZOL and FLC:ZOL on membrane fluidity (Fig. 4A) (32). In all four strains, treatment with FLC:ZOL resulted in a 1.88- to 2.48-fold decrease in anisotropy, indicating significantly reduced membrane rigidity (*P* range =0.0068–0.0500). Treatment with ZOL alone resulted in a significant decrease in rigidity for *C. albicans* and *C. glabrata* (*P* range =0.0083–0.0406) but not *C. krusei* (*P* = 0.0775). Membrane fluidity in cells treated with FLC, ZOL, and FLC:ZOL was similar to each other, with no significant difference observed between treatments in any of the four strains tested (*P* range =0.3187–>0.9999).

Membrane rigidity and ergosterol content have been previously linked to the activity of active transporters in the plasma membrane in *Candida* (33). Due to the capacity of *C. glabrata* to upregulate membrane-bound ABC transporters in response to drugs, the effects of ZOL and FLC:ZOL on active efflux rates were measured using rhodamine 6G, a fluorescent ABC transporter substrate (Fig. 4B). Treatment with FLC at MIC_c_ increased the maximum efflux rate in *C. albicans, C. glabrata* CBS138 (568 ΔRFU/sec), and *C. glabrata* M494893 (858 ΔRFU/sec) but not *C. krusei,* where FLC resistance is due to reduced binding affinity of Erg11 to drugs and not ABC transporters. In *C. albicans*, there was a 3.04-fold difference between the maximum efflux rates of FLC- and FLC:ZOL-treated cells (*P* = 0.0254). This difference was 3.96-fold in FLC-susceptible *C. glabrata* strain CBS138 (*P* = 0.0013), increasing to a massive 15.05-fold difference in FLC-resistant *C. glabrata* strain M494893 (*P* = 0.0005). It appeared that for *C. albicans* and *C. glabrata,* FLC:ZOL treatment inhibited active transport to such an extent that the rate of passive diffusion of rhodamine 6G into the cell exceeded the rate of efflux, resulting in negative efflux rates. This was quite different from VPM, a known efflux pump inhibitor, where efflux was reduced but not reversed.

### ZOL and FLC:ZOL have species-specific effects on the permeability of *Candida* membranes

**Fig 5:**
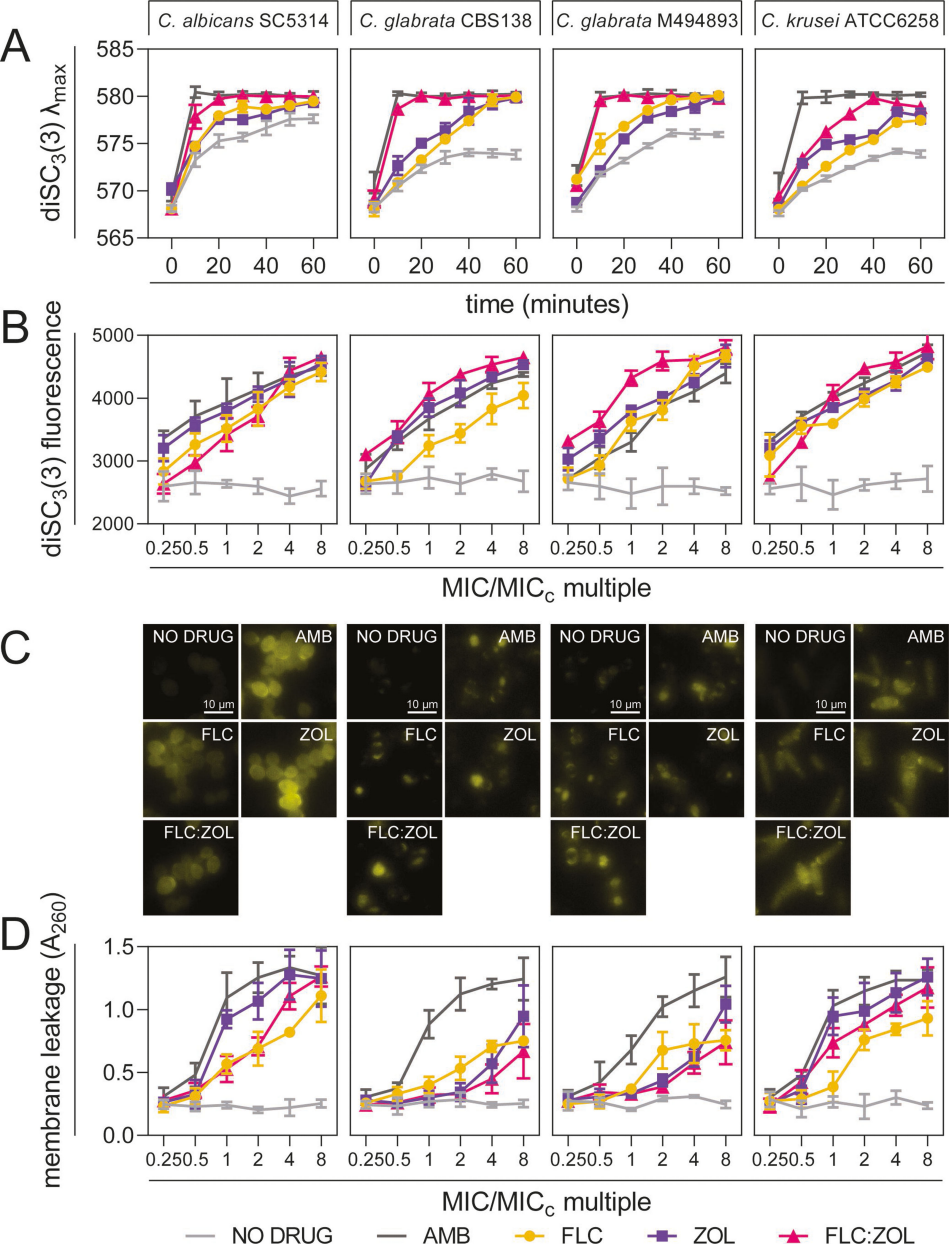
ZOL and FLC:ZOL induce depolarization and permeabilization of the yeast plasma membrane. (**A**) Changing membrane depolarization over time for *Candida* cells treated with a no-drug control (1% DMSO) (light gray lines), FLC at MIC (yellow circles), ZOL at MIC (purple squares), FLC:ZOL at MIC_c_ (pink triangles), and AMB at MIC (dark gray lines), determined by diS-C_3_(3) maximal emission wavelength (λ_max_). (**B**) Dose-dependent membrane depolarization of *Candida* cells treated with increasing levels of FLC at MIC (yellow circles), ZOL at MIC (purple squares), FLC:ZOL at MIC_c_ (pink triangles), a no-drug control (1% DMSO) (light gray lines), and AMB at MIC (dark gray lines), determined by diS-C_3_(3) fluorescence intensity. (**C**) Fluorescence microscopy of diS-C_3_(3)-stained *Candida* cells treated with the indicated drugs for 1 hour. Images were obtained at an exposure time of 100 ms. (**D**) Membrane permeabilization, assessed by the level of nucleic acids in the supernatant of *Candida* cultures. Cells were treated with the indicated concentrations of a no-drug control (1% DMSO) (light gray lines), FLC at MIC (yellow circles), ZOL at MIC (purple squares), FLC:ZOL at MIC_c_ (pink triangles), and AMB at MIC (dark gray lines). Data presented are the means of three biological replicates ± SD.

DiS-C_3_(3) is a fluorescent probe that accumulates in yeasts with depolarized membranes, causing it to red-shift and increase in fluorescence intensity (34). AMB is known to cause membrane depolarization and was used as a positive control, showing rapid increases in the λ_max_ of DiS-C_3_(3) for all strains (Fig. 5A). FLC:ZOL treatment caused the rapid depolarization of *C. albicans* and *C. glabrata* membranes but had a more limited effect in *C. krusei*. In all four strains, FLC:ZOL caused more rapid changes in membrane polarity than either FLC or ZOL alone. Changes in fluorescence intensity (Fig. 5B and C) at 1× MIC/MIC_c_ reveal that FLC, ZOL, and FLC:ZOL all have deleterious effects on membrane polarity. Figure 5B shows that FLC, ZOL, and FLC:ZOL induced membrane depolarization in a dose-dependent manner (*r* = 0.8918, 0.8658, and 0.8153, respectively).

Membrane permeabilization was assessed by measuring the leakage of nucleic acids into the cell supernatant (Fig. 5D) (35). Both ZOL and FLC:ZOL had dose-dependent effects on membrane leakage in *C. albicans* (*r* = 0.9351 and 0.9383, respectively), *C. glabrata* CBS138 (*r* = 0.9987 and 0.9832), *C. glabrata* M494893 (*r* = 0.9935 and 0.9955), and *C. krusei* (*r* = 0.8618 and 0.8489). Despite their higher sensitivity, the two *C. glabrata* strains showed significantly less membrane leakage than *C. albicans* (*P* = 0.0038 and 0.0139, respectively) or *C. krusei* (*P* = 0.0075 and 0.0259) when treated with ZOL alone and when treated with FLC:ZOL (*C. albicans P* = 0.0032 and 0.0210; *C. krusei P* = 0.0008 and 0.0063, respectively).

### Bisphosphonate–azole synergy causes oxidative stress in *Candida glabrata*

**Fig 6:**
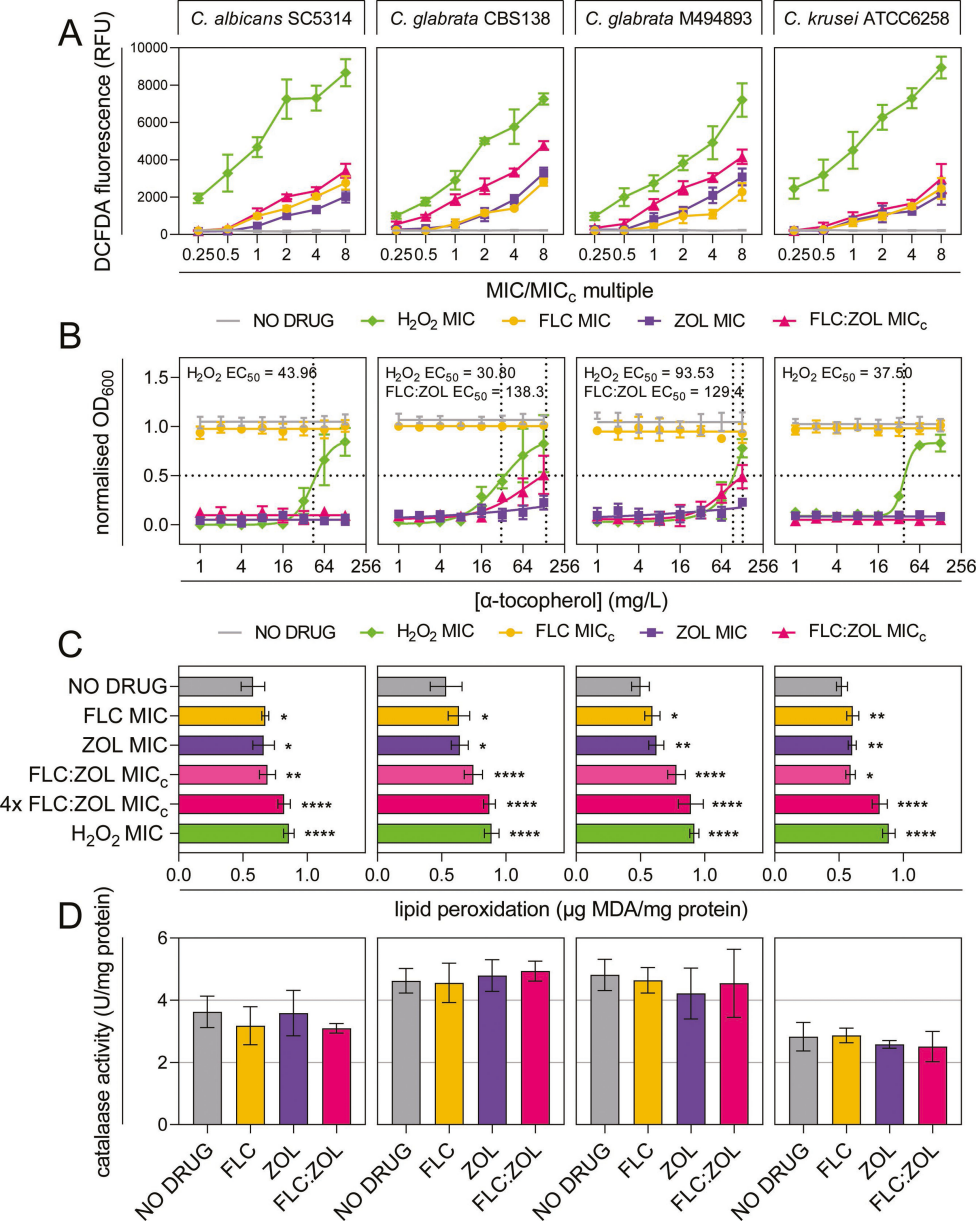
FLC:ZOL synergy causes accumulation of intracellular ROS and oxidative stress in *Candida glabrata* but not in *C. albicans* or *C. krusei.* (**A**) Generation of ROS in *Candida* cells treated with a no-drug control (1% DMSO) (gray lines), FLC (yellow circles), ZOL (purple squares), FLC:ZOL (pink triangles), and H_2_O_2_ (green diamonds) assessed by DCFDA fluorescence. (**B**) Antioxidant rescue using α-tocopherol on *Candida* cultures treated with a no-drug control (1% DMSO) (gray lines), FLC at MIC_c_ (yellow circles), ZOL at MIC (purple squares), FLC:ZOL at MIC_c_ (pink triangles), and H_2_O_2_ at MIC (green diamonds). (**C**) Lipid peroxidation in drug-treated *Candida,* assessed by the peroxidation of malondialdehyde (MDA) normalized to total protein content in cell lysates. All treatments were compared to the no-drug control (1% DMSO) by one-way ANOVA and found to be statistically significant. (**D**) Catalase activity, assessed by measuring the rate of decomposition of H_2_O_2_ normalized to total protein content, in cell lysates of *Candida* cultures treated with a no-drug control (1% DMSO), FLC or ZOL at MIC, and FLC:ZOL at MIC_c_. For (A–C), data presented are the mean of two technical replicates from each of three biological replicates ± SD; for (D), data are the mean of three technical replicates from each of three biological replicates ± SD. Treatments were compared to the DMSO control by one-way ANOVA.

The accumulation of intracellular reactive oxygen species (ROS) in drug-treated *Candida* was determined by measuring the fluorescence intensity of dichlorodihydrofluorescein diacetate (DCFDA) (Fig. 6A). MICs for H_2_O_2_, used as a positive control throughout this section, are detailed in Table S4. For all strains, treatment with FLC, ZOL, and FLC:ZOL caused a dose-dependent increase in intracellular ROS (*r* = 0.9857, 0.9851, and 0.9507, respectively). FLC:ZOL at 1× MIC_c_ caused significantly more ROS accumulation than FLC and ZOL at MIC in *C. glabrata* CBS138 (*P* < 0.0001) and *C. glabrata* M494893 (*P* < 0.0001), but there was no significance for *C. albicans* or *C. krusei.* FLC:ZOL treatment also induced significantly more ROS in the two *C. glabrata* strains than in *C. albicans* (*P* < 0.0001) or *C. krusei* (*P* < 0.0001).

The addition of α-tocopherol, a potent antioxidant, reduced ROS-dependent killing by H_2_O_2_ in all strains and partially rescued the *C. glabrata* strains, with 128 µg/mL α-tocopherol restoring 53.40% growth of *C. glabrata* CBS138 and 55.59% growth of *C. glabrata* M494893. α-Tocopherol did not rescue *C. albicans* or *C. krusei* from FLC:ZOL treatment (Fig. 6B).

Lipid peroxidation was used as an indicator of oxidative damage in drug-treated cells (Fig. 6C). In all four strains, FLC and ZOL at 1× MIC caused minor but statistically significant increases in lipid peroxidation (*P* < 0.05). At 1× MIC_c_, FLC:ZOL caused significantly more peroxidation than FLC or ZOL in *C. glabrata* CBS138 (*P* = 0.0171 and 0.0273, respectively) and *C. glabrata* M494893 (*P* < 0.0001), but not *C. albicans* or *C. krusei.* Increasing FLC:ZOL to 4× MIC_c_ increased oxidative damage to a level similar to 1× MIC for H_2_O_2_ for *C. albicans* (*P* = 0.6156), *C. glabrata* CBS138 (*P* = 0.9891), and *C. glabrata* M494893 (*P* = 0.8500), but not for *C. krusei*, for which H_2_O_2_ was still significantly more damaging (*P* = 0.0073).

To determine if the observed increase in ROS accumulation in *C. glabrata* was due to differences in catalase expression, the effects of drug treatment on catalase activity in each of the four strains were measured. None of the treatments significantly affected catalase activity in any of the *Candida* strains (Fig. 6D). *Candida glabrata* is known to have a limited suite of iron uptake genes and only one siderophore transporter, and catalase expression is known to be demanding on available iron (36, 37). To determine if the sensitivity to ROS was due to differences in iron homeostasis, exogenous iron was added to ZOL and FLC:ZOL-treated *Candida*. This failed to rescue growth at any iron concentration (Fig. S1).

### FLC:ZOL rescues infected *Galleria mellonella* larvae at a lower dosage than FLC or ZOL alone

**Fig 7:**
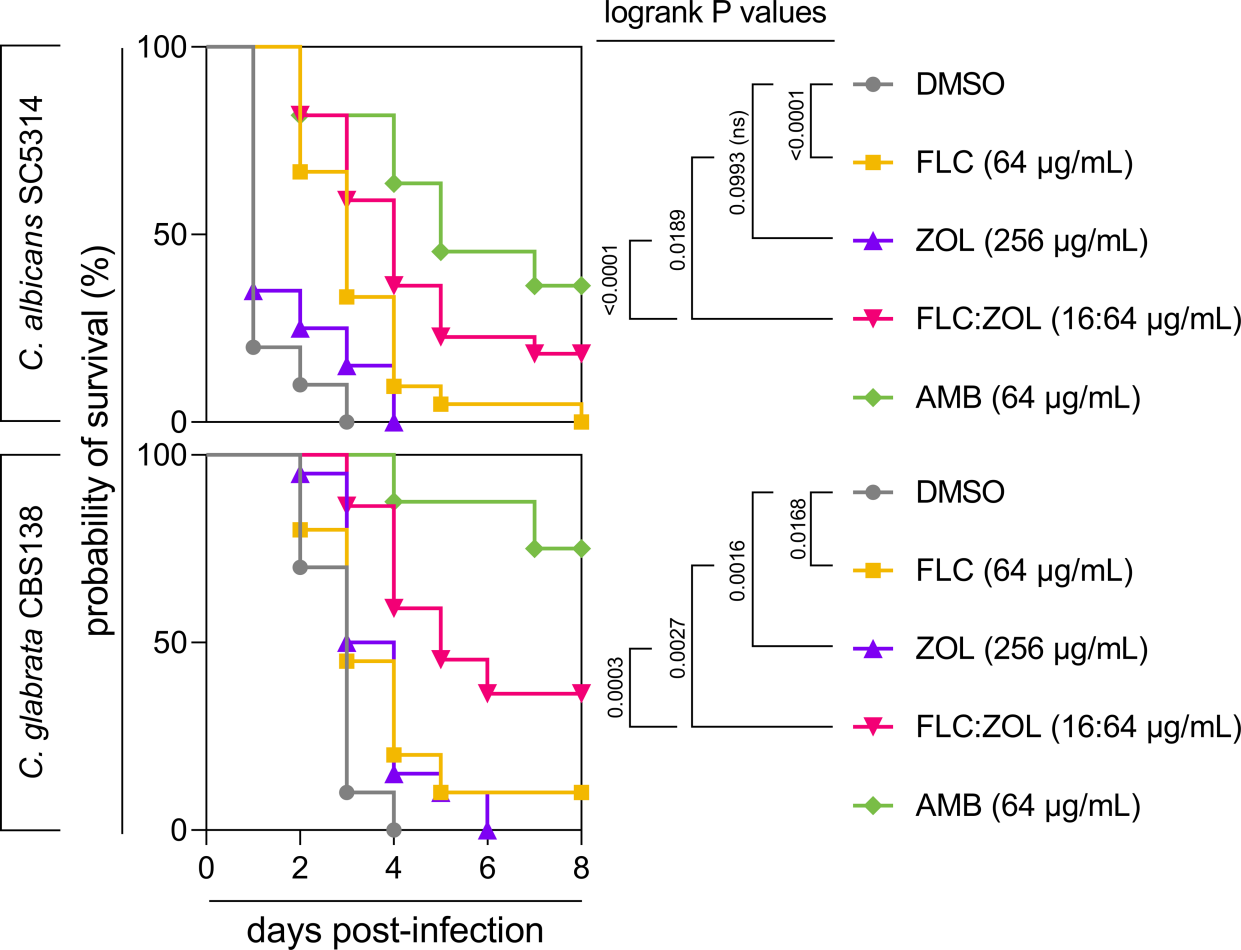
Combining FLC and ZOL improves the survival of *Galleria mellonella* larvae infected with *Candida. G. mellonella* larvae (*n* = 24) were injected with *C. albicans* SC5314 at 10^5^ cells/larva or *C. glabrata* CBS138 at 5 × 10^6^ cells/larva. Infected larvae were treated with a no-drug control (1% DMSO) (gray circles), FLC at 64 µg/mL (yellow squares), ZOL at 256 µg/mL (purple triangles), FLC:ZOL at 16:64 µg/mL (pink inverted triangles), or AMB at 64 µg/mL (green diamonds). Mortality was monitored over 8 days. Kaplan–Meier plots represent two independent experiments.

The *in vivo* efficacy of FLC:ZOL combination therapy was tested in an invertebrate model of infection using *C. albicans* and *C. glabrata* CBS138. A combination of FLC:ZOL (16:64 µg/mL) improved the survival of *C. albicans*-infected larvae over FLC (64 µg/mL; *P* = 0.0189) or ZOL (256 µg/mL; *P* < 0.0001) alone (Fig. 7). Similarly, for *C. glabrata,* FLC:ZOL was more effective than FLC (*P* = 0.0027) or ZOL (*P* = 0.0003) alone. At 8 days post-infection, the FLC:ZOL combination therapy rescued 20% of *C. albicans-*infected larvae and 40% of *C. glabrata-*infected larvae.

## DISCUSSION

**Fig 8:**
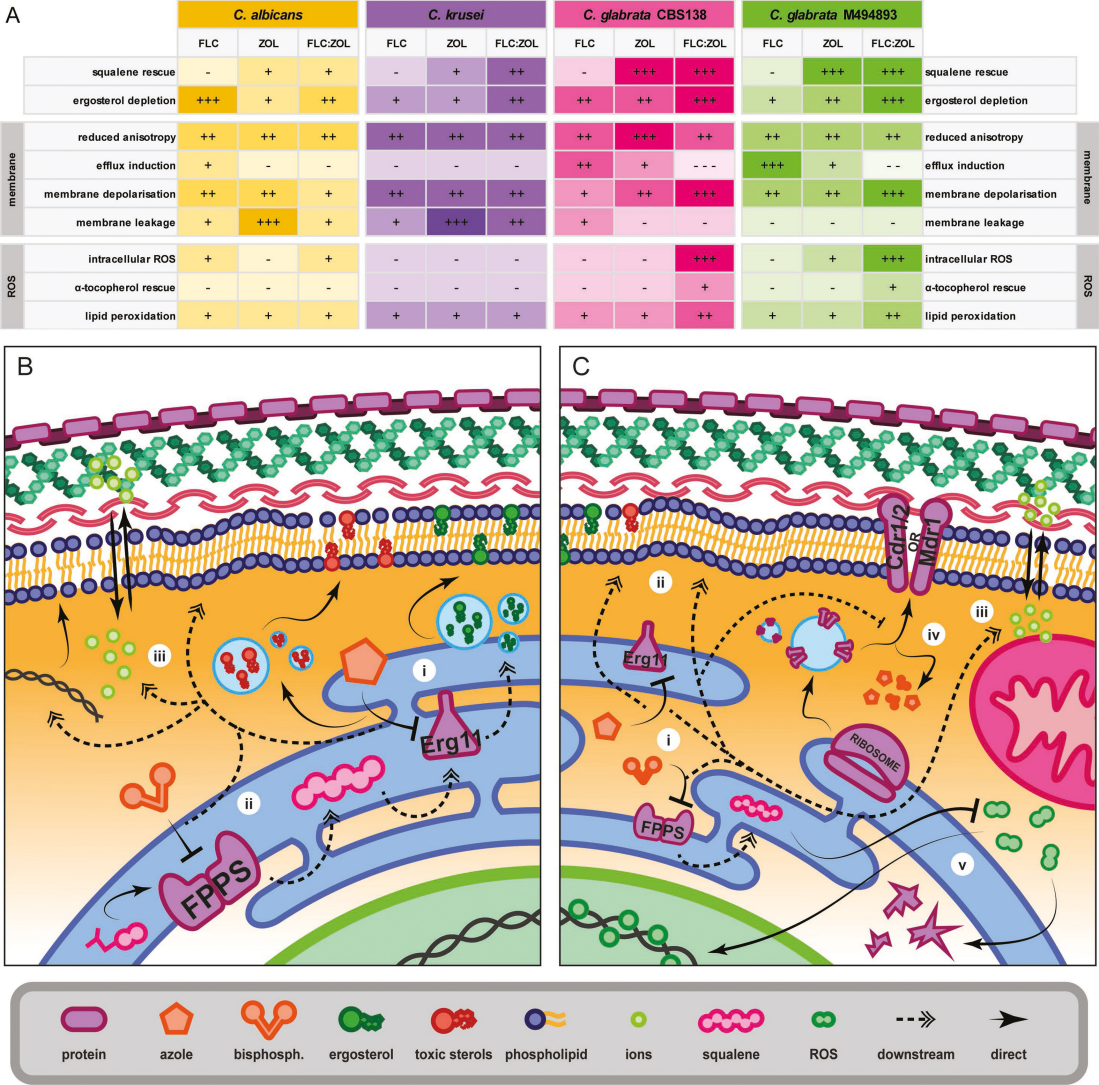
A proposed model for the antifungal mechanism of bisphosphonate–azole synergy in *C. albicans, C. krusei, and C. glabrata*. (**A**) Table summarizing the relative response of *C. albicans*, *C. krusei*, fluconazole-sensitive *C. glabrata* (CBS138), and fluconazole-resistant *C. glabrata* (M494893) to FLC, ZOL, and FLC:ZOL across the various assays performed in this study. (**B**) Proposed mechanism of azole–bisphosphonate synergy in *C. albicans* and *C. krusei*. Here, azoles inhibit Erg11, preventing the demethylation of lanosterol and causing the accumulation of toxic ergosterol precursors in the membrane (i). The addition of bisphosphonates inhibits FPPS, preventing the synthesis of squalene (ii), and this critically depletes membrane ergosterol. This results in a reduced membrane structure, including hyperfluidity and depolarization, causing nucleic acids and other vital cell components to leak into the cell supernatant (iii). (**C**) Proposed mechanism of azole–bisphosphonate synergy in *C. glabrata*. Although azoles and bisphosphonates cause critical ergosterol depletion (i) similar to that described for *C. albicans* and *C. krusei* resulting in membrane hyperfluidity (ii) and depolarization (iii), the major impact in *C. glabrata* is dysfunction and mis-localization of membrane-bound active efflux pumps (iv). This impairs the expulsion of antifungals, resulting in an increased intracellular concentration and enhanced toxic effect. FLC:ZOL treatment causes a higher intracellular concentration of ROS in *C. glabrata* that contributes to oxidative damage and cell death (v).

In this study, we have extended our previous work on azole–bisphosphonate synergy in *Cryptococcus* by applying these drug combinations to a range of species of *Candida*. We found broad synergy between azoles and bisphosphonates but also identified species-specific differences in susceptibility. *C. glabrata* was particularly susceptible to most azole–bisphosphonate combinations, which was initially surprising given the high levels of azole resistance in some of the *C. glabrata* isolates. However, further mechanistic studies revealed that the active efflux that mediates azole resistance in *C. glabrata* was profoundly disrupted by the depletion of ergosterol and subsequent compromised membrane structure. Based on the results of our analyses (summarized in Fig. 8A), we propose two separate mechanisms of azole–bisphosphonate synergy for different species of *Candida* (Fig. 8B and C) and discuss this further below.

### Combining bisphosphonates and azole antifungals results in critical depletion of ergosterol, but the effect of this differs between *Candida* species

The inhibition of squalene synthesis results in the depletion of membrane ergosterol, which is critical to the antifungal mechanism of bisphosphonates and becomes profoundly toxic when combined with azoles. This was confirmed by supplementation with exogenous squalene, which completely negated the antifungal effects of ZOL and FLC:ZOL (Fig. 8). Squalene is an intermediate in the biosynthesis of ergosterol, which is a vital fungal membrane lipid responsible for the regulation of membrane structure, the formation of lipid rafts, and the localization and function of transmembrane proteins (38). Squalene biosynthesis is, therefore, a potentially attractive antifungal target; however, to date, it has not been widely explored. Statins inhibit squalene synthesis upstream from FPPS at the point of mevalonate synthesis and have been found to synergize with azoles in certain fungal species (39). However, supplementing statin-treated *C. albicans* with ergosterol failed to mitigate antifungal activity, indicating that ergosterol depletion is not the key to their mechanism of action (40). Previous research has suggested that squalene itself can directly restore the rigidity of hyperfluid membranes by forming small lipid droplets that have a stabilizing effect in ergosterol-deprived *Saccharomyces cerevisiae,* which may partially account for its ability to rescue growth in ZOL- and FLC:ZOL-treated cells (41). However, the direct incorporation of squalene is a double-edged sword, as the accumulation of squalene in healthy membranes results in permeabilization, which is integral to the antifungal activity of terbinafine (42). In our study, the complete rescue of viability by squalene supplementation suggests that FLC:ZOL combinations directly target the mevalonate and ergosterol pathways of *Candida* (Fig. 8B and C), although the downstream consequences of synergy differ significantly between species.

Ergosterol depletion due to FLC:ZOL synergy had a drastic effect on drug efflux and resulted in irreversible oxidative damage in *C. glabrata.* All strains of *Candida glabrata* were hypersensitive to ZOL and FLC:ZOL synergy despite a very high level of azole resistance in some. While bisphosphonates caused catastrophic membrane damage resulting in leakage and cell death in *C. albicans* and *C. krusei* (Fig. 8B)*,* we propose a unique mechanism of FLC:ZOL synergy in *C. glabrata* that is summarized in Fig. 8C. Although it is well established that drugs and other toxins stimulate pump activity in *C. glabrata*, which was seen here for FLC and ZOL when used independently, the FLC:ZOL combination significantly downregulated active efflux. Cdr1, an important drug efflux transporter, is known to require a regulated membrane structure and ergosterol-rich lipid rafts to translocate to the membrane and function properly in *S. cerevisiae* and *C. albicans* (33, 43, 44). It is, therefore, likely that the negative effect of the azole–bisphosphonate combination on the sterol composition and rigidity of the cell membrane inhibited efflux, resulting in the accumulation of azoles and bisphosphonates inside the cell. This caused yet more inhibition of ergosterol biosynthesis and membrane compromise in a feedback loop that led to hypersensitivity. *C. glabrata* also showed an accumulation of ROS, with rescue by α-tocopherol suggesting that this participated in cell death. Previous research has found that *C. glabrata* has a more robust response to oxidative stress than other *Candida* species, likely due to its ability to upregulate catalase (45). However, *C. glabrata* is unable to express the membrane-bound transporters for glutathione that most fungal pathogens, including *C. albicans* and *C. krusei,* use to scavenge oxidants and ameliorate oxidative stress and instead must synthesize glutathione *de novo* (46, 47). This possibly caused the increased ROS lethality observed in *C. glabrata*.

### Bisphosphonates are promising lead compounds for the treatment of candidemia

Azoles and bisphosphonates are promising combination therapies as they are highly synergistic in a comprehensive collection of *Candida* isolates. FLC:ZOL was investigated thoroughly in this study; however, combinations containing ALN and RIS were also largely synergistic. This contrasts with our prior research on fluconazole–bisphosphonate combinations in *Cryptococcus*, where ZOL was the only bisphosphonate able to reliably synergize with FLC (27). Antifungal applications of ALN for candidiasis may be worth investigating further as well; ALN has more favorable oral pharmacokinetics and can be administered more frequently than ZOL due to its lower toxicity, and it may, therefore, be suitable in other therapeutic contexts (48, 49). Azole–bisphosphonate combinations were remarkably effective in most species of *Candida*, even in highly azole-resistant strains, which indicates that bisphosphonates may be useful for reducing effective dosages of azoles and overcoming acquired resistance.

This study is the first to report antifungal synergy in *Candida* through the inhibition of FPPS (Erg20); however, other attempts have been made to synergistically increase the activity of azoles by targeting closely related pathways (19). Erg251 and Erg3, both upstream of Erg20, have been explored as targets for combination antifungal therapy; however, while some synergy was observed, it was highly strain- and species-specific in both *Cryptococcus* and *Candida* (50, 51). As stated above, statins also inhibit the mevalonate pathway and have demonstrated some antifungal synergy with azoles, albeit in a comparatively narrow array of isolates (39). Retrospective clinical studies have found that patients taking statins to control cholesterol positively correlated with a reduced risk of developing candidiasis and showed that diabetic adults who take statins while undergoing treatment for candidiasis required shorter periods of antifungal therapy (52). Statin intake during treatment for candidemia also correlated with significantly reduced mortality in multiple cohort studies (53, 54). It would be of great interest to perform a similar retrospective study for patients on long-term bisphosphonate therapy and determine if this also correlates with positive clinical outcomes for preventing or alleviating fungal infections.

### Azole–bisphosphonate combinations are promising for the treatment of superficial candidiasis and could be further developed for invasive infection

After further pharmacological development, on-market bisphosphonates could potentially be repositioned as synergists for the treatment of mucocutaneous candidiasis, which presents an enormous clinical burden and is responsible for millions of hospital visits per year (1). Although invasive infections rarely stem from true superficial infections, they are frequently initiated by the colonization and dysbiosis of *Candida* on the surface of mucosal tissues (3, 55). Cream- or ointment-based topical azole–bisphosphonate therapies could be developed to both reduce oropharyngeal and vulvovaginal infections and prevent their development into disseminated infections. Candidemia frequently follows colonization of implanted devices with *Candida* biofilms that are refractory to treatment (3), and the antibiofilm activity of the azole–bisphosphonate combinations could reduce this complication, which has become a leading cause of nosocomial infections (56). Our demonstration that bisphosphonates and azole–bisphosphonate combinations were effective across a range of non-*albicans* species and were especially effective in *C. glabrata* is particularly promising as these are increasingly responsible for oral and vaginal symptoms that are either recurrent or refractory to treatment (8, 57). Finally, the development of resistance that is common following repeated or prophylactic use of azoles may be mitigated by bisphosphonate synergy (58).

Although they are exciting lead compounds, there are limitations to the usefulness of current bisphosphonates in systemic antifungal therapy. While bisphosphonates are relatively non-toxic and well tolerated in long-term therapy and were effective at resolving *Candida* infection in *Galleria mellonella*, their strong binding affinity for bone mineral means that their bioavailability is severely reduced in peripheral tissues, where the burden of fungal infection is often the highest. To overcome these limitations, lipophilic zoledronate derivatives have been developed, where a lipid tail of 1–15 carbon residues was covalently attached to a nitrogen moiety. Derivatives with a 10-carbon alkyl tail exhibited reduced bone binding and improved bioactivity, demonstrating excellent *in vitro* and *in vivo* efficacy in the treatment of malaria and trypanosomiasis, and these compounds should be investigated for antifungal activity (59, 60). Another potential limitation is the finding that *C. glabrata* may be able to overcome ergosterol depletion by replacing it with cholesterol from the host serum (61) and may resist azoles by upregulating sterol uptake genes (62). These studies each investigated a single *C. glabrata* isolate, and it remains to be demonstrated if this is common in *C. glabrata* infections. In our study, the azole–bisphosphonate combinations severely inhibit active efflux via membrane disruption, and it is feasible that they also inhibit the localization and function of membrane-bound sterol transporters. Understanding the true efficacy of azole–bisphosphonate therapy requires its application in an *in vivo* murine model of candidemia.

### Conclusion

This study has explored the therapeutic potential of azole–bisphosphonate therapy as a treatment of candidiasis. A major advantage of this therapy is that it repurposes already-approved compounds, which may significantly expedite the drug development pipeline (20). We have demonstrated broad-spectrum synergy between azoles and bisphosphonates in a range of *Candida* species. Bisphosphonates act on the mevalonate pathway, resulting in the depletion of squalene and membrane ergosterol and causing a dysregulated membrane structure. In *Candida glabrata*, this causes dysfunction of active efflux pumps and oxidative stress, while in other *Candida* species, the antifungal effects appear to be caused by damage to the plasma membrane and cell leakage. We have shown that these combinations are effective at resolving infection in a *G. mellonella in vivo* model and, upon further development, may be promising agents for mucocutaneous candidiasis. Bisphosphonates could be chemically modified to improve activity and decrease bone binding for invasive *Candida* infections.

## MATERIALS AND METHODS

### *Candida* strains

Forty-six clinical isolates of various *Candida* species were used in this study. Of those isolates, 11 are of the species *C. albicans*, 11 are *C. glabrata,* 6 are *C. parapsilosis*, 5 are *C. krusei*, 7 are *C. tropicalis,* and 6 are *C. dubliniensis*. The strain name and source of each isolate are detailed in Table S1.

### Susceptibility and synergy

Antifungal susceptibility was determined by broth microdilution according to the CLSI guidelines described in M27-Ed4 (28). Briefly, freshly grown colonies were taken from Sabouraud dextrose agar (SDA) plates after 48 hours of incubation at 30°C and suspended in phosphate-buffered saline (PBS). Cells were counted and diluted in RPMI-1640 (Sigma-Aldrich) with 165 mM MOPS and 2% dextrose to obtain a final inoculum of approximately 1 × 10^3^ cfu/mL. The maximum test concentrations were 256 µg/mL for FLC, RIS, ALN, and ZOL and 16 µg/mL for ITR and KET. MIC_80_ was read visually for FLC, and MIC_100_ was read visually for all other agents.

MFCs were determined by back-plating drug-treated cultures from MIC experiments onto SDA plates and incubating them for 48 hours at 30°C. The MFC was defined as the lowest drug concentration from which no colonies could be grown. To calculate the MICs and MFCs in this study, the mode of three biological replicates was calculated. For FLC, RIS, ALN, and ZOL, an MIC or MFC >256 µg/mL was assigned a value of 512 µg/mL. For ITR and KET, MICs > 16 µg/mL were assigned a value of 32 to enable the calculation of synergy.

The synergy between azole antifungals and bisphosphonates was determined by a checkerboard assay using the Loewe additivity model (29). Two-dimensional serial dilutions were prepared in 96-well microtiter plates for each azole and bisphosphonate pair, starting at two times the MIC listed.. The lowest MIC for each individual drug when combined (MIC_c_) was determined visually. FICI was calculated as the sum of the ratios between the MIC_c_ and the MIC of each drug. Any combination with an FICI ≤ 0.5 was considered synergistic. FICIs were calculated as the means of three biological replicates. Selected checkerboards were analyzed spectrophotometrically using a BioTek ELx800 plate reader to determine synergy by Bliss Independence using MacSynergy II (30). FFCIs were determined by back-plating checkerboard assays onto SDA plates.

### Antifungals and bisphosphonates

Stock solutions of FLC, ITR, KET, and AMB (Sapphire Bioscience) were prepared according to the CLSI standard M27-Ed4 for antifungal susceptibility testing (28). Stock solutions of RIS and ALN (Sigma-Aldrich) were prepared in water, and solutions of ZOL (Sigma-Aldrich) were prepared in 0.1 N NaOH, all at 5.12 mg/mL. Solvent concentrations were kept constant across dilutions during susceptibility testing to control for any antimicrobial effects. One percent of dimethyl sulfoxide (DMSO) was used as a no-drug solvent control throughout this study.

### Time-kill assays

Inocula of 10^6^ cells of *C. albicans* SC5314, *C. glabrata* CBS138, *C. parapsilosis* ATCC22018, *C. krusei* ATCC6258, *C. tropicalis* M230640, and *C. dubliniensis* M230642 were aliquoted into 10 mL of yeast peptone dextrose (YPD) broth containing a 1% DMSO control, FLC and ZOL at the MIC (Table S1), and combined FLC:ZOL at one, two, and four times the MIC_c_ (Table S2). Cultures were incubated at 37°C with shaking at 200 rpm. At 3, 6, 12, 24, and 48 hours post-inoculation, 100 µL aliquots were withdrawn, serially diluted in PBS, and back-plated onto SDA. Colonies were counted after 48 hours of incubation at 30°C to determine viable cell density. Four technical replicate plates were counted for each experiment, and three biological replicates were performed.

### Biofilm inhibition

The inhibition of mature biofilms produced by *C. albicans* SC5314, *C. glabrata* CBS138, *C. parapsilosis* ATCC22018, *C. krusei* ATCC6258, *C. tropicalis* M230640, and *C. dubliniensis* M230642 was investigated using the XTT reduction assay (63). Briefly, overnight broth cultures of each strain were counted and adjusted to 1 × 10^6^ cells/mL in RPMI-1640; then, 100 µL of the cell suspension was transferred into 96-well microtiter plates. Plates were then incubated for 24 hours at 37°C, the media was aspirated, and mature biofilms were washed three times with PBS to remove non-adherent planktonic yeasts. Serial twofold dilutions starting at 1,024 µg/mL solutions were prepared for each azole and bisphosphonate in RPMI-1640, and 200 µL of each concentration was added to the biofilms. After a further 24 hours of incubation at 37°C, 100 µL of XTT solution (500 µg/mL XTT, 1 µM menadione) was added to each well. Plates were incubated for 4 hours; then, 75 µL of the supernatant was transferred to a new plate and read spectrophotometrically at 490 nm in a BioTek ELx800 plate reader. The SMIC_80_ was determined as the antifungal concentration where there was an 80% decrease in absorbance compared to untreated biofilms. Mature biofilms were also treated with azoles and bisphosphonates in a checkerboard to determine the SFICI. The SMIC_c_ for each drug in combination was determined as described above, and the sum of the ratios of the SMIC_c_ and the SMIC of each drug was calculated to give the SFICI. SMIC_80_s were calculated as the mode of three replicates, and SFICIs were calculated as the means of the three replicates.

### Induction of antifungal resistance

To investigate whether combining FLC and ZOL prevents the development of resistance to either compound, antifungal resistance induction experiments were performed as described previously, with slight modifications (27). Inocula of 10^3^ cells from a shaking overnight broth cultures of *C. albicans* SC5314, *C. glabrata* CBS138, *C. parapsilosis* ATCC22018, *C. krusei* ATCC6258, *C. tropicalis* M230640, and *C. dubliniensis* M230642 were counted and aliquoted into YPD broth containing either a 1% DMSO control, FLC or ZOL at 0.25× MIC, or FLC:ZOL at 0.25× MIC_c_. Cultures were incubated at 37°C with shaking at 200 rpm for 24 hours, and viability was determined by back-plating a 100 µL aliquot onto SDA plates and counting colonies. A 100 µL aliquot of each culture was taken, then subcultured into YPD broth and incubated at 37°C with shaking overnight. From this overnight culture, 10^3^ cells were counted and subcultured into YPD broth containing either a DMSO control, FLC or ZOL at 0.5× MIC, or FLC:ZOL at 0.5× MIC_c_. The method was repeated for gradually increasing concentrations of FLC, ZOL, and FLC:ZOL. Four technical replicate plates were counted for each treatment, and three biological replicate experiments were performed.

### Squalene rescue assays

Mechanistic experiments were performed on *C. albicans* SC5314, azole-sensitive *C. glabrata* CBS138, azole-resistant *C. glabrata* M494893, and *C. krusei* ATCC 6258. The FLC and ZOL MICs and FLC:ZOL MIC_c_s used in these experiments are detailed in Table 3. AMB was used as a positive control for experiments on membrane activity, and H_2_O_2_ was used as a positive control for oxidative stress. The dosages used for these are also listed in Table 3.

The rescue of cultures treated with zoledronate was performed as described previously (27). Squalene (Sigma-Aldrich) was diluted in acetone to 25.6 mg/mL, diluted 1:100 in RPMI-1640, and then serially diluted to achieve a maximum final test concentration of 256 µg/mL and a minimum concentration of 0.0625 µg/mL. ZOL was added according to the MIC of each strain (Table S1), and FLC and FLC:ZOL were added at MIC_c_ (Table S2). 1 × 10^3^ cells/mL from fresh overnight cultures were inoculated into RPMI-1640 in 96-well titer plates containing the relevant compounds. Optical density at 600 nm (OD_600_) was read spectrophotometrically in a BioTek ELx800 plate reader after 24 hours at 35°C. Growth was normalized to a no-inoculum control and a no-treatment control, and non-linear regression analysis was performed to obtain a dose–response curve and calculate the effective concentration of squalene that restores 50% of inhibited growth (EC_50_). Three independent biological replicates were performed, each with two technical replicates.

### Ergosterol quantitation

Cell membrane ergosterol was quantified as described previously with slight modifications (64). Cultures were grown in an overnight shaking broth culture; cells were pelleted, washed twice with PBS, and adjusted to 10^5^ cells/mL in 10 mL of YPD broth containing either DMSO, FLC and ZOL at the MIC and the MIC_c_, and FLC:ZOL at 1× and 4× the MIC_c_. Cultures were treated with either 128 µg/mL exogenous squalene or an acetone solvent control. Treated cultures were incubated at 30°C for 18 hours with shaking at 200 rpm. Cells were pelleted and washed with Milli-Q water, and the cell pellet was weighed. Three milliliters of 25% KOH in EtOH was added to each pellet and vortexed for 1 minute, and the resulting lysate was transferred to a borosilicate screw-cap tube. Lysates were incubated in an 80°C water bath for 1 hour and then cooled. One milliliter of Milli-Q water and 3 mL of *n-*heptane were added to the tubes and vortexed vigorously for 3 minutes. The organic phase was transferred to a fresh screw-cap tube and stored at −30°C overnight. Two hundred microliters of extracts was diluted in 800 µL 100% EtOH, and absorbance at 230 and 281.5 nm was measured in a UV-1600PC spectrophotometer (VWR). The percent ergosterol was calculated as [(5(*A*_281.5_ ÷ 290)) ÷ pellet weight] – [(5(*A*_230_ ÷ 518)) ÷ pellet weight], where 5 is the dilution factor of extracts in ethanol and 290 and 518 are the extinction coefficients for ergosterol and dihydroergosterol. Three biological replicates were performed.

### Membrane fluidity

Effects of bisphosphonate treatment on the membrane fluidity of *Candida* were investigated using steady-state anisotropy of TMA-DPH (32). Overnight cultures were pelleted, washed twice with PBS, and adjusted to 1 × 10^5^ cells/mL in 2 mL of YPD broth containing a DMSO control, FLC and ZOL at MIC (Table S1), or FLC:ZOL at MIC_c_ (Table S2). Cells were treated for 6 hours in a 12-well plate (Corning), pelleted and washed twice with PBS, and then labeled with 2 µM TMA-DPH in PBS. Cells were incubated in the dark for 10 minutes; then, 200 µL was transferred to optically clear 96-well microplates (Invitrogen). Parallel and perpendicular fluorescence intensities were measured at 440 nm in a CLARIOstar plate reader (BMG Labtech), and anisotropy was calculated as (parallel – perpendicular) ÷ (parallel + 2(perpendicular)). Three independent biological replicates were performed, each with two technical replicates.

### Active efflux assays

The effect of bisphosphonates on the function of membrane-bound active efflux pumps was determined as described previously with slight modifications (65). Cells from an overnight culture were pelleted and washed twice in Milli-Q water before being resuspended and diluted to 10^5^ cells/mL in 10 mL YP broth (20 g/L peptone, 10 g/L yeast extract) containing FLC, ZOL, and FLC:ZOL at MIC_c_, a DMSO control, or 2 mg/mL VPM, an efflux pump inhibitor acting as a positive control. After shaking at 200 rpm at 30°C for 4 hours, cells were pelleted and resuspended in YP containing the appropriate drug and 20 µM rhodamine 6G (Sigma Aldrich), a fluorescent efflux pump substrate. Cultures were incubated with shaking at 30°C for 30 minutes in the dark. Treated cells were pelleted, placed on ice and washed twice with refrigerated YP broth, and then resuspended with 10 mL warm YPD broth containing the appropriate drug, with continued incubation in the dark. Every 12 minutes, 2 × 200 µL aliquots were transferred to fresh microfuge tubes, and cells were pelleted. One hundred microliters of the supernatant was transferred to an optically clear microplate (Invitrogen), and fluorescence intensity of rhodamine 6G in the supernatant was measured by excitation at 515 nm and emission at 555 nm in a CLARIOstar plate reader (BMG Labtech). Efflux rates were calculated as a change in relative fluorescence units per second over 12 minutes. Three independent biological replicates were performed, each with two technical replicates.

### Membrane depolarization assays

Depolarization of bisphosphonate-treated *Candida* membranes was measured with a potential-sensitive fluorescent probe, diS-C_3_(3) (Sigma-Aldrich), as described previously (66). Cells from overnight cultures were adjusted to 10^5^ cells/mL in 2 mL YPD broth containing 1% DMSO (no-drug solvent control), FLC or ZOL at MIC, or FLC:ZOL at MIC_c_. AMB at MIC was included as a positive control. Treatments were incubated for 6 hours, then pelleted, washed twice with a citrate-phosphate (CP) buffer (82.5 mM Na-citrate, 17.5 mM citric acid, pH 6.0), and resuspended in 20 nM diS-C_3_(3) in CP buffer to a final concentration of 10^6^ cells/mL. Aliquots were then transferred to an optically clear 96-well microplate (Invitrogen). Emission spectra between 565 and 585 nm (λ_excitation_ = 531 nm) were obtained every 10 minutes for an hour in a CLARIOstar plate reader (BMG Labtech), and the λ_max_ was determined at each timepoint.

To determine a dose–response relationship between drug concentration and membrane depolarization, total diS-C_3_(3) fluorescence was also measured. Cells were prepared as described above but treated with a gradient of antifungal compounds between 0.25× and 8× the MIC for FLC, ZOL, and AMB; and MIC_c_ for FLC:ZOL. Fluorescence at 580 nm was measured with a CLARIOstar plate reader (BMG Labtech); then, cells from the 1× MIC/MIC_c_ treatments were transferred onto glass slides for examination with a Nikon Eclipse Ti fluorescence microscope (Nikon). For all membrane depolarization experiments, three independent biological replicates were performed, each with two technical replicates.

### Membrane permeabilization assays

Membrane permeability was investigated by measuring the passive release of nucleic acids into the supernatant of drug-treated *Candida* (35). Cells from overnight cultures were pelleted, washed twice with PBS, and diluted to 10^6^ cells/mL in 2 mL of Milli-Q water containing 0.25×–8× the MIC of FLC and ZOL (Table S1) or 0.25×–8× the MIC_c_ of FLC:ZOL. AMB at 0.25×–8× the MIC was included as a positive control. Cultures were incubated at 30°C with shaking at 200 rpm in a 12-well plate (Corning) for 6 hours. After treatment, cultures were passed through a 0.45 µm syringe filter, and the *A*_260_ of filtrates was measured with a DS-11 FX+ benchtop spectrofluorometer (DeNovix). Each reading was blanked with Milli-Q water containing the matching drug at the appropriate concentration. Three independent biological replicates were performed, each with three technical replicates.

### Quantifying intracellular ROS

The accumulation of intracellular ROS in *Candida* cells treated with bisphosphonates was investigated using DCFDA (Sigma-Aldrich), a ROS-sensitive fluorophore, as described previously with slight modifications (67). Cells from overnight and pre-stationary phase cultures were pelleted, washed twice with PBS, and adjusted to 10^5^ cells/mL in 2 mL YPD broth containing 0.25×–8× the MIC of FLC and ZOL (Table S1) and 0.25×–8× the MIC_c_ of FLC:ZOL (Table S2). H_2_O_2_ at 0.25×–8× the MIC was included as a positive control (Table S4). Cells were treated for 3 hours, pelleted, washed twice with PBS, and resuspended in PBS with 20 µM DCFDA. After 30 minutes of incubation at 30°C in the dark, cells were again pelleted, washed twice with PBS, and resuspended in 1 mL PBS. Three 200-µL aliquots of the cell suspensions were added to an optically clear microplate (Invitrogen), and the fluorescence intensity (λ_excitation_ = 485 nm, λ_emission_= 535 nm) was measured with a CLARIOstar plate reader (BMG Labtech). Three independent biological replicates were performed, each with three technical replicates.

### Lipid peroxidation

The thiobarbituric acid reactive substances (TBARS) assay was used to assess the extent of lipid peroxidation by ROS in bisphosphonate-treated *Candida* (68, 69). A TBARS assay kit (Cayman Chemical #700870) was used to measure the concentration of MDA, a by-product of lipid peroxidation, in cell lysates according to the manufacturer’s instructions. Briefly, overnight cultures of *Candida* cells were pelleted, washed twice with PBS, and resuspended in 10 mL of YPD broth containing a DMSO control, FLC and ZOL at MIC (Table S1), positive control H_2_O_2_ at MIC (Table S4), and FLC:ZOL at 1× and 4× MIC_c_ (Table S2). Cultures were incubated with shaking at 200 rpm at 30°C for 6 hours; then, cultures were pelleted and adjusted to 2 × 10^7^ cells in 1 mL of sterile ice-cold PBS. The cell suspensions were sonicated at 20 kHz for two 10-sec bursts with an Q2000 ultrasonicator (Qsonica). MDA was then extracted and quantitated according to the manufacturer’s instructions with a CLARIOstar plate reader (BMG Labtech). *A*_532_ was compared to the total protein content in the cell lysates. Extracts were analyzed in three technical replicates, and three independent biological replicates were performed.

### Tocopherol rescue assays

The role of ROS in the inhibition of *Candida* by FLC:ZOL synergy was further interrogated by attempting to rescue growth by supplementation with α-tocopherol, a potent antioxidant (70). α-Tocopherol (Sigma-Aldrich) was prepared at 128 µg/mL and serially diluted to 1 µg/mL in RPMI-1640. ZOL was added according to the MIC of each strain (Table S1), and FLC and FLC:ZOL were added at MIC_c_ (Table S2). H_2_O_2_ was included as a positive control at MIC (Table S4). 1 × 10^3^ cells/mL from fresh overnight cultures were inoculated into RPMI-1640 in 96-well titer plates containing the relevant compounds. OD_600_ was read in a CLARIOstar plate reader (BMG Labtech) after 24 hours of incubation at 30°C. Growth was normalized to a no-inoculum control and a no-treatment control, and non-linear regression analysis was performed to obtain a dose–response curve and calculate the effective concentration of α-tocopherol that restores 50% of inhibited growth (EC_50_). Three independent biological replicates were performed, each with three technical replicates.

### Catalase activity assays

The effect of azole and bisphosphonate treatment on *Candida* catalase activity was investigated as described previously, with minor modifications (71). Catalase activity of whole cell lysates was determined by measuring the rate of decomposition of H_2_O_2_, which absorbs at 240 nm. Cells from overnight cultures were pelleted, washed in sterile PBS, and resuspended at 10^5^ cells/mL in 10 mL YPD containing a DMSO control, FLC and ZOL at MIC (Table S1), and FLC:ZOL at MIC_c_ (Table S2). After 4 hours of treatment, cells were pelleted, washed with sterile phosphate buffer (PB) (20 mM KH_2_PO_4_, 40 mM Na_s_HPO_4_, pH 7.0) with complete protease inhibitors (Sigma-Aldrich, #11836145001), and resuspended in 500 µL PBS with protease inhibitors. Fifty microliters of 1 mm silica beads (Sigma-Aldrich) was added to the cell suspensions and was homogenized for three 30-sec bursts in a Minilys bead beater (Bertin). Cell debris and silica beads were removed by centrifugation, and the supernatant was transferred to a fresh tube and stored at −30°C overnight. Total protein content in cell lysates was quantified by diluting lysates 1:50 with sterile water, mixing a 5 µL aliquot with 250 µL Coomassie Blue (Sigma-Aldrich), and measuring the *A*_595_ in a CLARIOstar plate reader (BMG Labtech). Absorbances were converted into protein concentrations by comparison with a bovine serum albumin (BSA) standard (Sigma-Aldrich). One milliliter of diluted lysates was combined with 1 mL of PB and 1 mL of 30 mM H_2_O_2_ in a quartz cuvette, then mixed by inversion. After blanking with PB, the *A*_240_ was read every 30 sec for 2 minutes in a UV-1600PC spectrophotometer (VWR). The catalase activity was then calculated as described previously (72). Lysates were analyzed in three technical replicates, and three biological replicates were performed. Catalase activities of drug-treated *Candida* were compared to the DMSO control by one-way analysis of variance (ANOVA).

### *Galleria mellonella* infections

*G. mellonella* larvae were reared in an environmentally controlled room at Macquarie University, Sydney, Australia, at 30°C and 65% humidity with a 12-hour light/dark cycle. The injections were performed as previously described (73). Briefly, overnight cultures of *C. albicans* SC5314 and *C. glabrata* CBS138 were grown in 5 mL YPD broth at 30°C, pelleted, washed twice, and resuspended in PBS. Larvae (200–250 mg) were individually injected with 10 µL of cell suspension into the last right pro-leg using a 100 µL syringe (Hamilton Ltd.). Larvae were inoculated with *C. albicans* SC5314 and *C. glabrata* CBS138 at 10^5^ and 5 × 10^6^ cells per larva, respectively. Two hours post-infection, 10 µL of FLC at 64 µg/mL, ZOL at 256 µg/mL, FLC:ZOL at 16:64 µg/mL, or an AMB positive control (64 µg/mL) in PBS was injected into the last left pro-leg. The ZOL dosage was chosen as higher concentrations were found to be toxic to *G. mellonella* larvae in preliminary testing. The FLC:ZOL dosage was chosen as it is a fourfold decrease in the initial dosage of each drug individually, which is the cut-off for synergy according to the Loewe additivity model. Sterile PBS with 1% DMSO was also injected as a no-treatment control. Following injection, the larvae were incubated at 37°C and monitored every 24 hours for 8 days. Biological duplicate experiments were performed (*n* = 12 larvae per treatment).

### Statistical analysis

MICs, MFCs, FICIs, and SFICIs were compared by one-way ANOVA. Correlations between azole susceptibility and bisphosphonate susceptibility were determined by Pearson correlation coefficient, *r.* Comparisons of membrane ergosterol content, anisotropy, depolarization and permeabilization, ROS accumulation, and lipid peroxidation were made using one-way ANOVA. Maximum efflux rates were compared using Welch’s *t*-test. The dose dependence of membrane depolarization, leaked nucleic acids, and accumulation of ROS was determined by linear regression analysis. *G. mellonella* Kaplan–Meier survival curves were compared using the logrank *P*-test.

## SUPPLEMENTAL MATERIAL

Figure S1 spectrum.00121-24-s0001.docx Exogenous iron does not rescue bisphosphonate-mediated inhibition of *Candida*.

Supplemental tables spectrum.00121-24-s0002.xlsx Tables S1-S4.

ASM does not own the copyrights to Supplemental Material that may be linked to, or accessed through, an article. The authors have granted ASM a non-exclusive, world-wide license to publish the Supplemental Material files. Please contact the corresponding author directly for reuse.
