# Supplementary material for: Spectrum of activity and mechanisms of azole–bisphosphonate synergy in pathogenic Candida
Source: Microbiol Spectr. 2024 May 2;12(6):e00121-24. doi: 10.1128/spectrum.00121-24 (PMC11237636; doi:10.1128/spectrum.00121-24)
Supplement: Figure S1 — Exogenous iron does not rescue bisphosphonate-mediated inhibition of Candida. [file spectrum.00121-24-s0001.docx]

**
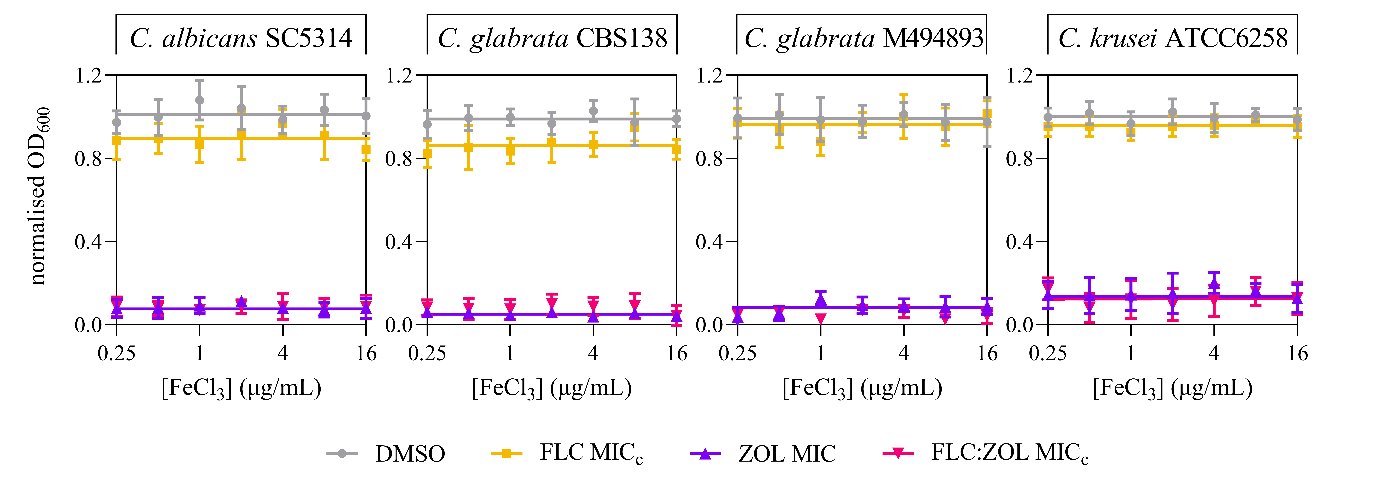
**

**Supplementary Figure S1. Exogenous iron does not rescue bisphosphonate-mediated inhibition of *Candida*.**
